# Digital compassion fatigue and digital empathy in healthcare: mapping the evidence and informing a human-centered clinical framework

**DOI:** 10.3389/fpsyg.2026.1959352

**Published:** 2026-09-14

**Authors:** Ebtsam Aly Abou Hashish

**Affiliations:** 1 College of Nursing—Jeddah, King Saud bin Abdulaziz University for Health Sciences, Jeddah, Saudi Arabia; 2 King Abdullah International Medical Research Center, Jeddah, Saudi Arabia; 3 Ministry of National Guard Health Affairs, Jeddah, Saudi Arabia; 4 Faculty of Nursing, Alexandria University, Alexandria, Egypt

**Keywords:** artificial intelligence, compassionate care, Digital Compassion Fatigue, digital empathy, digital health, healthcare workforce, human-centered healthcare, scoping review

## Abstract

**Background:**

Digital transformation has fundamentally reshaped healthcare delivery, creating new opportunities for communication, care coordination, and clinical decision-making while introducing emotional and relational challenges for healthcare professionals. Among these challenges, Digital Compassion Fatigue (DCF) and Digital Empathy have emerged as closely related but conceptually distinct constructs. Despite increasing interest in both concepts, their theoretical relationship, defining characteristics, and implications for healthcare practice have not previously been synthesized within a unified framework.

**Objective:**

To synthesize the emerging literature on Digital Compassion Fatigue and Digital Empathy, map the current evidence base, identify conceptual and measurement gaps, and develop an evidence-informed Human-Centered Clinical Framework to guide research, education, leadership, and digitally mediated healthcare practice.

**Methods:**

This scoping review followed the Joanna Briggs Institute methodology and was reported according to the PRISMA Extension for Scoping Reviews (PRISMA-ScR). Eligibility criteria were based on the Population–Concept–Context (PCC) framework. Evidence sources were identified through comprehensive searches of six electronic databases, supplemented by Google Scholar, backward reference-list screening, and forward citation tracking. Eligible evidence sources were charted and synthesized using descriptive and narrative approaches.

**Results:**

The review included 41 evidence sources spanning concept analyses, empirical studies, qualitative studies, scoping reviews, systematic reviews, meta-analyses, and theoretical or framework papers. Digital Compassion Fatigue (DCF) remained at an early conceptual stage, with no validated DCF-specific instrument identified, whereas Digital Empathy was supported by a broader conceptual and empirical literature. Because the evidence was heterogeneous and largely observational, conceptual, or secondary, the synthesis supports associations and conceptual propositions rather than causal pathways. The mapped evidence informed a testable Human-Centered Clinical conceptual model integrating digital demands, adaptive resources, organizational support, Digital Empathy, DCF, and patient, professional, and system outcomes.

**Conclusion:**

This review integrates two previously separate bodies of literature and clarifies Digital Empathy and DCF as related but non-oppositional constructs within digitally mediated healthcare. The proposed Human-Centered Clinical model is evidence-informed rather than empirically validated and should be tested across professions, healthcare systems, and cultural contexts before it is used to support causal or clinical claims.

## 1 Introduction

Digital transformation has fundamentally reshaped healthcare delivery by changing how healthcare professionals communicate, document care, make clinical decisions, educate patients, and sustain therapeutic relationships (Abou Hashish and Alnajjar, 2025; Abou Hashish, 2025; Alotaibi et al., 2025). Electronic health records, telehealth platforms, mobile health applications, clinical decision-support systems, remote patient monitoring, and artificial intelligence are now embedded across most healthcare settings. Their adoption accelerated further once the COVID-19 pandemic normalized virtual consultation as a routine mode of care delivery (Bouabida et al., 2022). These technologies have expanded the reach, continuity, and efficiency of healthcare delivery. They allow geographically isolated or mobility-limited patients to access specialist input, allow care teams to coordinate across settings in real time. At the same time, this transformation does not simply add a new channel through which existing care is delivered; it changes the interpersonal and emotional conditions under which care itself is enacted. In digitally mediated encounters, clinicians must convey concern, recognize emotional cues, and preserve therapeutic presence despite physical distance, reduced non-verbal information, screen-mediated interaction, asynchronous communication, and continuously increasing technological demands on their attention. Digital transformation therefore affects not only how care is delivered, but also how compassion itself is expressed, perceived, sustained, and—as this review will argue—potentially depleted (Abou Hashish and Alnajjar, 2025; Abou Hashish, 2025).

Compassion and empathy are not incidental to healthcare; they are among its foundational commitments. Empathic communication supports shared understanding between clinician and patient, buffers emotional distress, strengthens therapeutic alliance, improves patient participation in care decisions, and is consistently associated with better-perceived quality of care (Abou Hashish, 2025; Abou Hashish and Atalla, 2023; Bail et al., 2023; Mercer et al., 2004). Compassion extends beyond recognizing another person's distress by incorporating the motivation to respond with humane and helpful action, distinguishing compassionate care from technical competence alone (Gilbert, 2009). Critically, the increasing digitization of clinical work does not remove the need for this relational foundation—f anything, it raises the stakes, because the channels that now carry so much of the clinical relationship (a portal message, a video consultation, a remote-monitoring alert) carry comparatively little of the non-verbal and contextual information clinicians have traditionally relied on to express and perceive compassion (Luetke Lanfer et al., 2024; Duffy et al., 2023). The central question is how compassion is preserved, adapted, or depleted when care is mediated through screens, messages, and algorithms.

One response to this question has been the emergence of the concept of *Digital Empathy*. The term has been in circulation since at least 2016, when it was introduced in health-professions education as the capacity to extend empathic communication skills into technology-mediated encounters (Terry and Cain, 2016). It has since been substantially developed within nursing scholarship, where it is conceptualized as a multidimensional capability involving authenticity, trust-building, effective digital communication, emotional engagement, adaptability, technological proficiency, and cultural sensitivity, with antecedents in digital literacy, emotional intelligence, and supportive technological infrastructure (Abou Hashish, 2025). An earlier framework for designing digital health tools with empathy had already emphasized engaging, entrusting, encouraging, and empowering users through technology, anticipating several of these same attributes before the term “digital empathy” had fully entered nursing scholarship (Abou Hashish, 2026).

Rather than representing a simple extension of bedside communication into virtual environments, Digital Empathy has emerged as a distinct adaptive competency in its own right. Clinicians must learn to interpret digitally conveyed emotional cues—a flat affect on video, for instance—to communicate attention and understanding through a medium that strips away many non-verbal signals, and to maintain professional authenticity when interaction is structured by devices, interfaces, and sometimes algorithms rather than by the room the clinician and patient share (Abou Hashish, 2025). A 2026 nursing-focused scoping review, drawing on a considerably broader evidence base of 33 studies published between 2009 and 2026, reached a convergent conclusion: digital empathy is best understood as a context-dependent extension of traditional empathy that adapts nursing's relational foundations to technology-mediated care, rather than a wholly new phenomenon or a lesser substitute for in-person empathy (Yildirim et al., 2026).

The digital qualifier therefore denotes a change in the conditions under which empathy is enacted rather than a wholly different human capacity. In face-to-face encounters, clinicians can draw on co-presence, touch, fuller body language, immediate conversational repair, and a shared physical environment. Digitally mediated encounters may reduce or transform these cues, alter eye contact, introduce latency and interface interruptions, and require emotion to be interpreted through text, audio, video, or algorithmically structured communication. Digital competence, platform usability, and access therefore become part of the conditions through which empathic intent is translated into perceived empathic care. For Digital Empathy, the current evidence supports this interpretation as a context-specific adaptation of empathy (Abou Hashish, 2025; Luetke Lanfer et al., 2024; Duffy et al., 2023; Terry and Cain, 2016; Yildirim et al., 2026). For DCF, however, current evidence does not establish a fundamentally separate syndrome from traditional compassion fatigue. Rather, DCF is best treated at this stage as a proposed technology-shaped form of compassion-related depletion in which sustained digital demands may modify how established fatigue processes are experienced and expressed (Abou Hashish and Alnajjar, 2025; Byrne, 2025; Maslach and Jackson, 1981; Figley, 2002; Peters, 2018; Sinclair et al., 2017).

A second, more recent and considerably less developed response has been the proposal of *Digital Compassion Fatigue (DCF)*. Digital Empathy describes an adaptive capability, whereas DCF describes a proposed form of depletion arising from sustained digital emotional demands. Technology-intensive care is known to generate measurable emotional and cognitive burden through channels that have little directly to do with empathy. Electronic-health-record design and use factors—information overload, slow system response, and after-hours documentation—have been repeatedly and robustly associated with clinician stress and burnout (Kroth et al., 2019; Hilliard et al., 2020), and a systematic review of 29 studies confirmed that usability issues and time spent in the record system were the strongest predictors of that association (Alobayli et al., 2023). Large systematic reviews and meta-analyses have consistently demonstrated associations between EHR burden and clinician burnout (Wu et al., 2024).

A parallel technostress and videoconference-fatigue literature independently links digital workload to burnout and reduced empathy among healthcare professionals (Bouabida et al., 2022; Bail et al., 2023; Kasemy et al., 2022; Nadler, 2020; Fauville et al., 2021; Simbula et al., 2023; Myronuk, 2022); Section 3.6 examines this evidence in detail. None of this, however, is quite the same as DCF. A recent concept analysis using Walker and Avant's method defined DCF specifically as a state of sustained emotional exhaustion and empathic depletion experienced by clinicians who deliver continuous, screen-mediated care, characterized by six attributes: emotional numbing in digital care, persistent post-engagement exhaustion, compassion withdrawal, cognitive overload from screen-mediated empathy, professional inefficacy in virtual environments, and diminished therapeutic presence (Abou Hashish and Alnajjar, 2025).

This built on an earlier evolutionary concept analysis that first proposed DCF as a technology-specific form of compassion fatigue among nurses experiencing technostress (Byrne, 2025). DCF should not be treated as interchangeable with burnout, traditional compassion fatigue, secondary traumatic stress, or technostress: burnout reflects chronic occupational strain involving exhaustion, cynicism, and reduced efficacy (Maslach and Jackson, 1981); compassion fatigue is more closely tied to the cumulative emotional cost of caring for people who suffer (Figley, 2002; Peters, 2018; Sinclair et al., 2017); technostress centers specifically on difficulty coping with technology demands (Wu et al., 2024; Kasemy et al., 2022); DCF is proposed to arise where technology-related burden intersects with sustained empathic and compassionate engagement specifically (Abou Hashish and Alnajjar, 2025; Byrne, 2025). Considerable conceptual overlap nonetheless remains, and the boundaries among these constructs are not yet well established empirically (Abou Hashish and Alnajjar, 2025).

Considered together, Digital Empathy and Digital Compassion Fatigue can be examined as related responses arising within the same digitally mediated clinical environment. The included evidence directly supports the existence of digital demands, technology-related fatigue, digitally mediated empathic practices, and associations between workload, usability, burnout, and relational outcomes. The proposition that Digital Empathy represents an adaptive pathway and DCF a maladaptive pathway is an integrative interpretation developed from this review, not a causal relationship demonstrated by the included studies. Digital Empathy may coexist with emotional exhaustion, and current evidence does not establish that higher Digital Empathy prevents DCF or that digital demands cause DCF.

Conversely, well-designed technology, manageable digital workload, psychological safety, and adequate training may sustain empathic digital practice while actively protecting professional wellbeing. Artificial intelligence sharpens this picture further, and the evidence here is genuinely double-edged rather than uniformly reassuring or alarming. Two large clinician-facing quality-improvement studies of AI-generated draft replies to patient portal messages found that generative AI could relieve documentation burden and supply an “empathy-infused draft” that helped clinicians overcome writer's block when responding to emotionally difficult messages (Tai-Seale et al., 2024; Garcia et al., 2024), and a systematic scoping review of artificial intelligence and compassion in healthcare similarly concluded that AI can support rather than replace compassionate communication when it is designed with that intent (Morrow et al., 2023).

The same body of evidence, together with qualitative work on digital clinical empathy in live-chat settings, also cautions that poorly designed or unreflectively deployed AI can create emotional distance, obscure professional responsibility, or encourage overreliance on automated responses (Luetke Lanfer et al., 2024; Morrow et al., 2023). Taken together, this suggests that Digital Empathy and DCF are best modeled not as a single axis but as two related, partly independent outcomes shaped by a common set of antecedents—the specific technologies in use, the digital workload they generate, the adaptive resources available to the individual clinician, and the organizational and design choices that govern how that technology is implemented.

Despite this conceptual convergence, the evidence relevant to Digital Empathy and DCF has developed largely in parallel rather than together, and it remains dispersed across nursing, medicine, psychology, telehealth, health informatics, and artificial intelligence research—much of it addressing adjacent phenomena without using either term explicitly. A coherent program of scoping-review work based at the University Health Network in Toronto has mapped compassion in digital health broadly (Wiljer et al., 2019), compassionate mental health care delivered digitally (Kemp et al., 2020), and—most recently—has proposed a modified-eDelphi interprofessional competency framework for what it terms “digital compassion” (Wiljer et al., 2025); a separate scoping review has mapped compassionate nursing care and digital health technologies specifically (Ali et al., 2022), and another has mapped compassion fatigue in virtual care (Crespo et al., 2023).

Complementary concept-analytic and narrative work has examined therapeutic relational connection in telehealth (Duffy et al., 2023), compassionate care delivered via eHealth (Su et al., 2025), the therapeutic alliance within digital mental health interventions (Tremain et al., 2020), and feelings of safety amid healthcare digitalization more broadly (Minartz et al., 2024), reinforcing that relational quality is a recurring concern across this literature even where DCF and Digital Empathy are not named directly. Most recently, and most directly adjacent to the present review, a 2026 scoping review mapped digital empathy in nursing in considerable methodological detail−33 included studies, JBI/PRISMA-ScR methodology, four databases—but did not address DCF at all and was restricted to a single profession (Yildirim et al., 2026).

Evidence on non-nursing professions delivering digitally mediated care exists but remains scattered rather than synthesized: psychologists reported worsening compassion fatigue and increased caseload intensity during pandemic-era telehealth delivery (Kercher et al., 2024), and teleworking psychologists showed elevated burnout across personal, work-related, and client-related domains on the Copenhagen Burnout Inventory (Serrão et al., 2022), yet no review has brought this multidisciplinary evidence together with the DCF and Digital Empathy literatures specifically, and none has proposed an integrative framework linking the two constructs as complementary outcomes of the same digital environment rather than as separate topics.

A scoping review is the appropriate design to close this gap, because the field is conceptually emerging, multidisciplinary, and methodologically heterogeneous (Peters et al., 2025; Arksey and O'Malley, 2005; Levac et al., 2010): rather than testing the effectiveness of a single intervention, this review maps how the two concepts have been defined and studied, identifies their antecedents and outcomes, examines the professions, technologies, and settings represented, and clarifies the gaps that require further investigation. Building on the existing concept analyses of DCF (Abou Hashish and Alnajjar, 2025) and Digital Empathy (Abou Hashish, 2025), and explicitly extending beyond both the recent nursing-only mapping of digital empathy (Yildirim et al., 2026) and the Toronto group's compassion-in-digital-health research program (Wiljer et al., 2019; Kemp et al., 2020; Wiljer et al., 2025), this review aims to synthesize evidence across both concepts and, informed by that synthesis. Further, to develop an evidence-informed Human-Centered Clinical Framework explaining how digitally mediated healthcare environments may foster adaptive empathic engagement or contribute to compassion-related depletion across individual, technological, organizational, and system levels.

What this review adds. Existing reviews have mapped Digital compassion Empathy in nursing, compassion in digital health, compassionate digital mental healthcare, and fatigue in virtual care separately. The present review extends this literature by synthesizing Digital Empathy and DCF across professional groups, explicitly comparing them with adjacent constructs, separating empirical findings from conceptual propositions, mapping their measurement status, and translating the combined evidence into a testable cross-level conceptual model. Its contribution is therefore not simply the juxtaposition of two constructs, but clarification of their boundaries, shared digital context, evidence maturity, and researchable relationships (Yildirim et al., 2026; Wiljer et al., 2019; Kemp et al., 2020; Wiljer et al., 2025; Ali et al., 2022; Crespo et al., 2023).

### 1.1 Review objectives

Consistent with the Joanna Briggs Institute (JBI) methodology for scoping reviews, this review pursued four overarching objectives: *(1) conceptual mapping*, to examine how Digital Compassion Fatigue (DCF) and Digital Empathy have been defined, conceptualized, and theoretically grounded; *(2) evidence mapping*, to characterize the volume, distribution, populations, healthcare settings, digital technologies, and methodological characteristics of the existing literature; *(3) analytical synthesis*, to integrate evidence on antecedents, attributes, consequences, measurement approaches, and relationships with related constructs, including burnout, technostress, secondary traumatic stress, moral injury, and resilience, while examining the implications of artificial intelligence for compassionate healthcare; and *(4) knowledge translation*, to identify evidence gaps and develop an evidence-informed Human-Centered Clinical Framework to inform research, education, leadership, healthcare practice, technology design, and policy.

Specifically, this review aimed to:

1. Map the volume, characteristics, and geographical distribution of research on Digital Compassion Fatigue and Digital Empathy in healthcare.
2. Examine how both concepts have been defined, conceptualized, and operationalized across the literature.
3. Identify the healthcare professions, populations, settings, and digital technologies represented in existing evidence.
4. Synthesize reported antecedents, defining attributes, manifestations, moderators, consequences, and measurement approaches for both concepts.
5. Examine relationships between Digital Compassion Fatigue and Digital Empathy and related constructs, including burnout, technostress, secondary traumatic stress, moral injury, empathic distress, and resilience.
6. Identify individual, organizational, technological, and educational strategies proposed to strengthen Digital Empathy or mitigate Digital Compassion Fatigue.
7. Explore the influence of artificial intelligence and other emerging digital technologies on compassionate healthcare practice.
8. Identify current evidence gaps and priorities for future research.
9. Develop an evidence-informed Human-Centered Clinical Framework integrating the available evidence on Digital Compassion Fatigue and Digital Empathy.

### 1.2 Main review question

What is currently known about Digital Compassion Fatigue and Digital Empathy among healthcare professionals, and how can the available evidence inform compassionate, sustainable, and human-centered healthcare in digitally mediated practice?

## 2 Methods

### 2.1 Study design

This review was designed as a scoping review because the objective was to map the breadth, characteristics, and conceptual maturity of an emerging, multidisciplinary evidence base rather than to test a specific hypothesis or estimate a pooled intervention effect. The review was conducted in accordance with the Joanna Briggs Institute (JBI) methodology for scoping reviews (Peters et al., 2025), building on the original framework proposed by Arksey and O'Malley (2005) and the methodological refinements described by Levac et al. (2010). The review is reported in accordance with the Preferred Reporting Items for Systematic Reviews and Meta-Analyses Extension for Scoping Reviews (PRISMA-ScR) (Tricco et al., 2018); the completed PRISMA-ScR checklist is provided as Supplementary File S1. The search strategy is documented following the PRISMA Search Reporting Extension (PRISMA-S) (Rethlefsen et al., 2021) to support transparency and reproducibility. The review question, eligibility criteria (Section 2.5), and planned analytic approach were nonetheless specified in advance of data charting and are reported in full in this manuscript and its Supplementary material.

### 2.2 Protocol and registration

No review protocol was prospectively registered. The review was conducted according to the Joanna Briggs Institute methodology for scoping reviews and reported in accordance with the PRISMA-ScR guideline.

### 2.3 Information sources

A systematic literature search was conducted across six electronic bibliographic databases: MEDLINE (via PubMed), CINAHL (EBSCOhost), PsycINFO (Ovid), Scopus, Web of Science Core Collection, and Embase (Ovid). These databases were selected to provide broad coverage of the biomedical, nursing and allied health, psychological, and multidisciplinary literature relevant to Digital Compassion Fatigue (DCF) and Digital Empathy. To maximize retrieval of eligible evidence, the electronic database searches were supplemented by Google Scholar, backward reference-list screening of all eligible studies, and forward citation tracking of the two anchor concept analyses (Abou Hashish and Alnajjar, 2025; Abou Hashish, 2025) and key review articles (Bouabida et al., 2022; Sinclair et al., 2017; Gustafsson and Hemberg, 2022; Labrague, 2021; Tai-Seale et al., 2024; Garcia et al., 2024).

All searches were conducted from database inception to 30 May 2026. The complete search strategies for each database are provided in Supplementary material 1.

### 2.4 Search strategy

The search strategy was developed to address the review question through three interconnected conceptual domains: (i) compassion- and empathy-related constructs, including Digital Empathy, virtual or online empathy, compassion fatigue, and technology-mediated compassion; (ii) digital healthcare technologies, including telehealth, electronic health records (EHRs), mobile health (mHealth), artificial intelligence (AI)-assisted care, and videoconferencing; and (iii) professional emotional and relational outcomes, including burnout, technostress, secondary traumatic stress, moral injury, and resilience.

To maintain conceptual specificity, eligible publications were required to address both compassion- or empathy-related constructs and digitally mediated healthcare. Terms describing professional emotional outcomes (e.g., burnout, technostress, resilience, and secondary traumatic stress) were used to increase search sensitivity within this intersection rather than as independent inclusion criteria, thereby minimizing retrieval of the broader burnout literature unrelated to compassionate digital care.

The complete search strategy, the full search strategies specified for all databases searched, and the documented search queries and citation-tracking procedures are provided in the Supplementary material to support transparency and reproducibility.

### 2.5 Eligibility criteria (population–concept–context)

Eligibility criteria were developed using the Population–Concept–Context (PCC) framework recommended by the JBI for scoping reviews.

#### 2.5.1 Population

Eligible sources included healthcare and social care professionals, including nurses, physicians, psychologists, counselors, social workers, allied health professionals, health-professions students, and interdisciplinary healthcare teams. Studies involving patients or informal caregivers were included only when their findings explicitly addressed healthcare professionals' empathy, compassion, emotional experiences, or wellbeing in digitally mediated care.

#### 2.5.2 Concept

The review focused on Digital Compassion Fatigue (DCF) and Digital Empathy, together with closely related concepts describing emotional and relational experiences within technology-mediated healthcare. These included technology-mediated empathy, virtual or online clinical empathy, telehealth empathy, electronic health record (EHR)-related emotional exhaustion, digital emotional labor, technostress, videoconference fatigue, technology-related empathic distress, digital resilience, and artificial intelligence (AI)-mediated compassionate communication. Sources were excluded if empathy or compassion fatigue was discussed without a meaningful connection to digital technologies or technology-intensive healthcare practice.

#### 2.5.3 Context

Eligible studies were conducted across healthcare and health-professions education settings, including hospitals, primary care, mental health services, rehabilitation, hospices, long-term care, community care, telehealth and virtual care, simulation-based education, EHR-supported practice, mobile health (mHealth), electronic health (eHealth), and AI-assisted healthcare. No restrictions were placed on geographical location or healthcare discipline. The complete inclusion and exclusion criteria are presented in Table 1.

**Table 1:** Eligibility criteria based on the PCC framework.

Domain	Inclusion criteria	Exclusion criteria
Population	Healthcare and social care professionals, clinical educators, health-professions students, interdisciplinary teams	Non-healthcare commercial, gaming, marketing, or general social-media populations
Concept	Digital Compassion Fatigue, Digital Empathy, technology-mediated empathy, EHR-related burnout/technostress, videoconference fatigue, digital emotional burden, related adaptive/maladaptive responses	Compassion fatigue, empathy, or burnout with no digital-health or technology-mediated component
Context	Clinical care, telehealth, EHRs, eHealth, mHealth, AI-assisted care, simulation, digital communication, health-professions education	Non-healthcare technology environments
Evidence type	Empirical studies, reviews, concept analyses, theoretical papers, frameworks, guidelines, authoritative reports	Conference abstracts, promotional material, news items, papers without substantive evidence
Language	English	Non-English
Time	Database inception to final search date	None based solely on publication year

### 2.6 Types of evidence sources

Eligible evidence sources included quantitative, qualitative, and mixed-methods studies; concept analyses; theoretical and framework papers; systematic, scoping, and integrative reviews; and authoritative professional guidelines or reports that contributed to understanding Digital Compassion Fatigue, Digital Empathy, or closely related concepts within digitally mediated healthcare. Conference abstracts, book reviews, editorials without substantive conceptual or empirical content, news articles, and other non-scholarly publications were excluded. Only English-language publications were included. No restriction was placed on publication year because both Digital Compassion Fatigue and Digital Empathy are recently emerging concepts, and earlier publications addressing related digital compassion or empathy constructs were considered potentially informative.

### 2.7 Selection process

Records identified through the electronic database searches and supplementary search methods were imported into Mendeley Reference Manager for reference management and duplicate removal. The deduplicated records were subsequently exported to Microsoft Excel, where title and abstract screening, eligibility assessment, and data charting were managed using predefined screening and extraction forms.

Following duplicate removal, records were screened against the predefined eligibility criteria (Section 2.5) through title and abstract review, followed by full-text assessment of potentially relevant publications. The study selection process is summarized in Figure 1.

**Figure 1:**
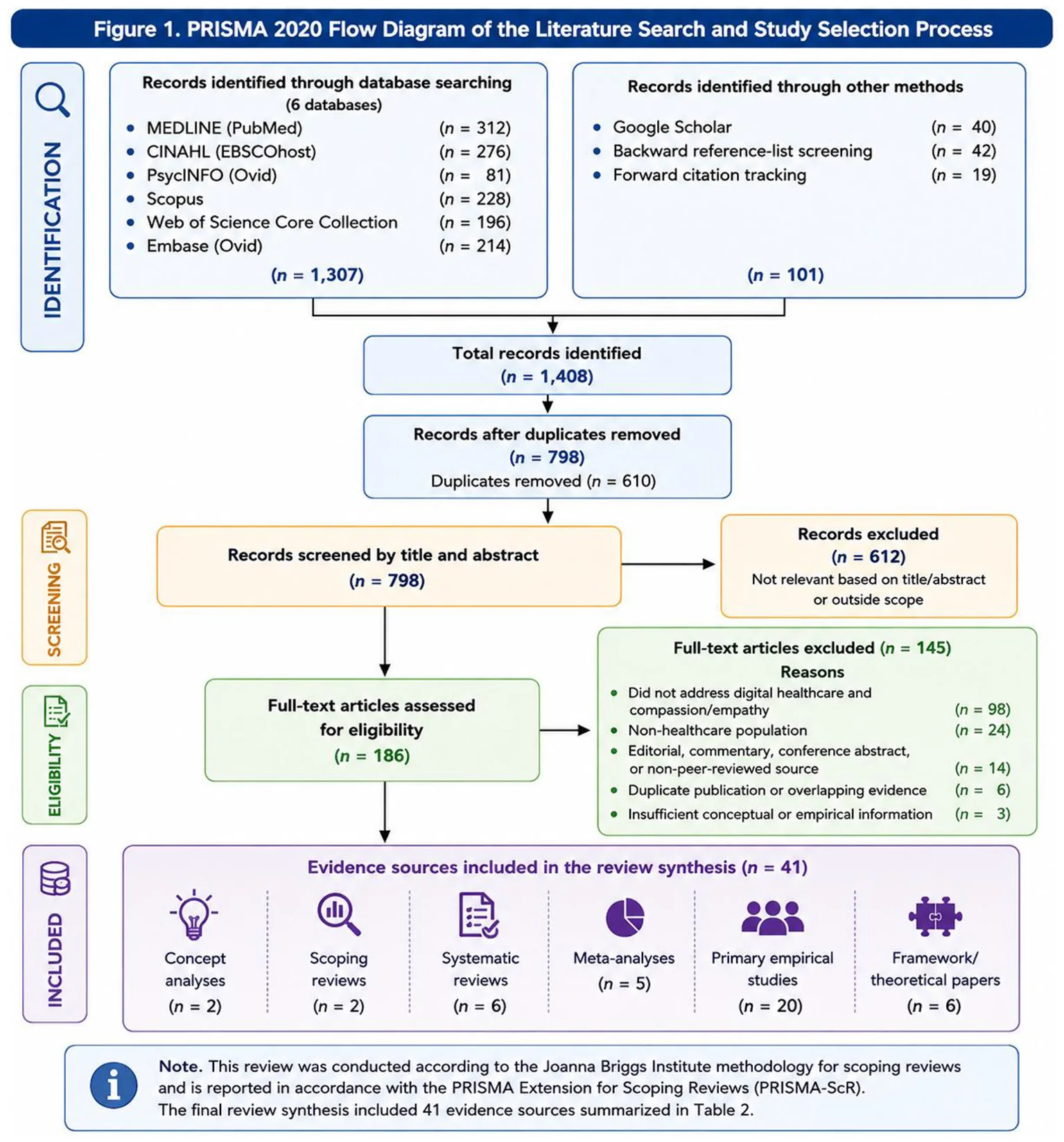
PRISMA flow diagram of the literature search and study selection process.

The literature search, study selection, and data charting were conducted by the first reviewer using the predefined eligibility criteria. A second reviewer independently verified the screening decisions, full-text eligibility assessment, and final selection of included evidence sources. Disagreements were documented in the screening file and resolved through discussion until consensus was reached. Because the second reviewer performed verification rather than duplicate independent screening of every record from the outset, an inter-rater agreement statistic was not calculated.

### 2.8 Data extraction

Data were extracted using a standardized data-charting form that was piloted on a sample of eligible evidence sources before full data extraction to ensure consistency and completeness. The extracted information included authorship, publication year, country, study design or evidence type, population or healthcare profession, healthcare setting, digital technology or clinical context, conceptual definitions, antecedents, defining attributes, outcomes or consequences, measurement approaches, and implications for research and practice. Additional information relating to theoretical foundations, relationships with adjacent constructs, and the role of artificial intelligence was extracted where reported. The complete data-charting variables are provided in Supplementary material 1. The extracted data informed the evidence mapping presented in Table 2 and the subsequent narrative synthesis.

**Table 2:** Characteristics of the included evidence informing the review synthesis.

Evidence source	Country	Study design	Population/setting	Digital technology/context	Primary concept(s)	Key contribution to the review
Concept analyses and conceptual foundations						
1. Abou Hashish and Alnajjar (2025)	Saudi Arabia	Concept analysis (Walker and Avant)	Nurses and clinicians in screen-mediated care	Digital and screen-mediated care	Digital Compassion Fatigue	Defined DCF and identified six attributes: emotional numbing, post-engagement exhaustion, compassion withdrawal, cognitive overload, professional inefficacy, and diminished therapeutic presence.
2. Abou Hashish (2025)	Saudi Arabia	Concept analysis (Walker and Avant)	Nursing and telehealth practice	Telehealth and digital communication	Digital Empathy	Defined Digital Empathy as a multidimensional capability involving authenticity, trust-building, communication, emotional engagement, adaptability, technological proficiency, and cultural sensitivity.
3. Terry and Cain (2016)	United States	Commentary	Pharmacy education	Digital communication	Digital Empathy	Provided the earliest identified use of the term Digital Empathy in health-professions education and argued for its inclusion in curricula.
4. Abou Hashish (2026)	International scope; published in Germany	Scholarly monograph	Nursing education, practice, leadership, and policy	Human-centered digital transformation	Human-centered digital transformation	Integrated theory, evidence, and practice on digital transformation in nursing and provided a theoretical foundation linking technology, leadership, adaptive capacity, compassionate digital care, and system outcomes.
5. Nadler (2020)	United States	Theoretical paper	Computer-mediated communication	Videoconferencing	Videoconference fatigue	Proposed the ‘third skin' explanation for fatigue arising from altered spatial and interactional demands in videoconferencing.
6. Byrne (2025)	United States	Evolutionary concept analysis	Registered nurses experiencing technostress	Digital and virtual platforms	Digital Compassion Fatigue	Positioned DCF as a technology-specific evolution of compassion fatigue linked to technostress in nursing.
7. Duffy et al. (2023)	United States	Concept analysis	Healthcare professionals	Telehealth	Therapeutic relational connection	Defined therapeutic relational connection in telehealth and clarified its antecedents, attributes, and consequences.
8. Gunawan (2026)	Not applicable	Theoretical and ontological paper	Nursing	AI and algorithmic care	Techno-caritas	Proposed an ontology linking digital empathy, caring, and algorithmic care in nursing.
9. Pepito et al. (2026)	Not applicable	Conceptual model development	Nursing	Technology-mediated care	Digital Empathy	Proposed a situated model explaining Digital Empathy in technology-mediated nursing environments.
Evidence syntheses and reviews						
10. Wiljer et al. (2019)	Canada	Scoping review protocol	Healthcare	Digital health	Compassion in digital health	Established a structured program for defining and mapping compassion in digital health.
11. Kemp et al. (2020)	Canada	Scoping review	Mental health professionals and services	Digital mental health technologies	Compassionate digital mental healthcare	Mapped strategies, facilitators, and barriers related to compassionate care in digital mental health.
12. Tremain et al. (2020)	Not reported	Narrative review	Digital mental health interventions	Digital mental health	Therapeutic alliance	Synthesized evidence on relational quality and therapeutic alliance in digital interventions for serious mental illness.
13. Ali et al. (2022)	Canada	Scoping review	Nurses	Digital health technologies	Compassionate nursing care	Showed that digital technologies can support compassionate nursing when implementation preserves relational care.
14. Crespo et al. (2023)	Spain	Scoping review	Healthcare professionals	Virtual care	Compassion fatigue	Mapped the emerging evidence linking compassion fatigue with virtual care delivery.
15. Morrow et al. (2023)	United Kingdom and international	Systematic scoping review	Healthcare professionals	Artificial intelligence	AI and compassion	Found that AI may support compassionate communication but that the relationship between AI and compassion remains under-conceptualized.
16. Alobayli et al. (2023)	United Kingdom	Systematic review	Hospital clinicians	Electronic health records	EHR-related stress and burnout	Identified EHR usability problems and time spent using EHRs as major contributors to clinician stress and burnout.
17. (Minartz et al. 2024)	Germany	Scoping review	Healthcare professionals and patients	Healthcare digitalization	Relational and psychological safety	Mapped how healthcare digitalization influences feelings of safety and relational experience.
18. Wu et al. (2024)	China	Systematic review and meta-analysis	Healthcare professionals; mainly physicians and nurses	Electronic health records	EHR-related burnout	Estimated pooled EHR-related burnout prevalence at 40.4% and found higher risk with after-hours EHR work.
19. Sarraf and Ghasempour (2025)	International scope	Systematic review	Healthcare professionals	Artificial intelligence and electronic health records	EHR-related burnout; AI	Synthesized evidence on how AI-related EHR functions may influence workload and burnout; contributes adjacent empirical context but does not operationalize DCF.
20. Howcroft et al. (2025)	United Kingdom	Systematic review and meta-analysis	Patients and healthcare professionals	AI chatbots	AI vs. human empathy	Found higher empathy ratings for AI chatbot responses than for human clinician responses in text-based interactions (pooled SMD 0.87).
21. Dall et al. (2025)	United Kingdom	Scoping review	Healthcare trainees	Serious and digital games	Digital empathy education	Found that digital games may support empathy development in healthcare education, although evidence remains limited.
22. Deriglazov et al. (2025)	Not reported	Systematic review and meta-analysis	Healthcare professionals	Mobile applications	Burnout, compassion fatigue, and compassion satisfaction	Synthesized mobile-app interventions targeting clinician well-being and compassion-related outcomes.
23. Yildirim et al. (2026)	Türkiye	Scoping review	Nurses; 33 studies published 2009-2026	Telehealth, telenursing, AI, and mHealth	Digital Empathy	Identified Digital Empathy as a context-dependent extension of traditional empathy across digitally mediated nursing care.
Primary empirical studies						
24. Kroth et al. (2019)	United States	Cross-sectional study	Primary care physicians	Electronic health records	Clinician stress and burnout	Linked specific EHR design and use factors with clinician stress and burnout.
25. Hilliard et al. (2020)	United States	Cross-sectional study	Clinicians	Electronic health records	EHR workload and burnout	Identified particular EHR activities and workload indicators associated with clinician burnout.
26. Kasemy et al. (2022)	Egypt	Multicenter cross-sectional study	Medical and nursing staff and students	Remote work and ICT	Technostress	Documented technostress creators and outcomes during COVID-19-related remote work.
27. Myronuk (2022)	Canada	Analytical perspective based on provider experience	Telemedicine clinicians	Videoconferencing	Provider fatigue and empathy	Argued that videoconferencing requires additional cognitive effort and may reduce provider empathy.
28. Serrão et al. (2022)	Portugal	Cross-sectional study	Psychologists	Teleworking	Burnout, depression, anxiety, and stress	Reported elevated personal, work-related, and client-related burnout among teleworking psychologists during COVID-19.
29. Tai-Seale et al. (2024)	United States	Quality-improvement study	Physicians	Generative AI for patient-message drafting	AI, workload, and empathic communication	Found that AI-generated drafts reduced documentation burden and provided empathy-oriented starting points for clinician responses.
30. Garcia et al. (2024)	United States	Quality-improvement study	Physicians	Generative AI for patient inbox messages	AI-assisted communication	Demonstrated the feasibility of AI-drafted replies and reported favorable empathy and readability ratings.
31. Luetke Lanfer et al. (2024)	Germany	Qualitative study and usability testing	Health professionals and patients	Live chat	Digital clinical empathy	Showed that empathic communication can be achieved in live-chat consultations and identified design and communication requirements.
32. Su et al. (2025)	Hong Kong, China	Qualitative study using interpretative phenomenological analysis	Nurses and physicians	eHealth, hotlines, apps, and social media	Compassionate care via eHealth	Described strategies and barriers experienced by clinicians when delivering compassionate care through eHealth.
33. Kercher et al. (2024)	New Zealand	Cross-sectional survey	Registered psychologists	Pandemic-era remote and telehealth practice	Compassion fatigue and professional quality of life	Reported increased work stress and caseload pressure alongside compassion fatigue and reduced resilience.
34. Steidtmann et al. (2024)	United States	Longitudinal survey	Academic mental health workforce	Videoconferencing	Burnout and videoconference fatigue	Found no association between videoconferencing and fatigue and observed lower burnout over time.
35. Muthukumar (2025)	United Kingdom	Cross-sectional rating study	Lay raters evaluating healthcare responses	ChatGPT and Claude	AI compassion	Found higher perceived compassion ratings for AI responses and identified response length as a predictor of perceived compassion.
36. Ruben et al. (2025)	United States	Experimental study	United States public evaluating online health-forum responses	ChatGPT and physician responses	AI empathy perception	Showed that perceived empathy depended on both the actual source and the source believed by participants.
37. Khan et al. (2025)	Pakistan	Cross-sectional study	Physicians	Prolonged virtual consultation and videoconferencing	Zoom fatigue	Linked cognitive load, reduced nonverbal cues, and interface stress with fatigue, anxiety, and dissatisfaction.
38. Khairat et al. (2025)	United States	Cross-sectional physiological study	Virtual nurses	Virtual nursing platforms	Cognitive fatigue	Used eye tracking and pupillometry to provide objective evidence of cognitive fatigue during virtual nursing workflows.
39. Everett et al. (2026)	United States	Randomized controlled trial	Clinicians (*n* = 70)	AI-supported diagnostic workflows	Clinician-AI collaboration	Showed that first-opinion vs. second-opinion AI workflow design influenced clinician performance and burden.
Competency and human-centered framework studies						
40. Wiljer et al. (2025)	Canada	Modified eDelphi consensus study	Interprofessional healthcare experts	Digital health environments	Digital compassion competencies	Developed a seven-domain interprofessional competency framework and identified technology attributes supporting digital compassion.
41. Girdwood et al. (2026)	United States	Perspective and framework paper	Oncology nurses and cancer-care navigation	Empathic and agentic AI	Human-centered AI in nursing	Proposed a human-centered framework for empathic and agentic AI in cancer-care navigation.

### 2.9 Data synthesis

The findings were synthesized using descriptive and narrative approaches, supported by tabular evidence mapping (Table 2) and graphical evidence visualization (Figures 1–5). Evidence sources were organized according to the review objectives to compare conceptual definitions, theoretical foundations, study characteristics, healthcare professions, clinical settings, digital technologies, antecedents, defining attributes, outcomes, and measurement approaches across the included evidence.

To preserve the evidentiary hierarchy within this heterogeneous scoping review, findings were classified during synthesis as: (1) primary empirical evidence, including quantitative, qualitative, mixed-methods, longitudinal, experimental, and quality-improvement studies; (2) secondary evidence, including systematic, scoping, and meta-analytic reviews; and (3) conceptual evidence, including concept analyses, theoretical papers, frameworks, commentaries, and scholarly models. Empirical associations were reported separately from conceptual propositions wherever possible. Conceptual and secondary sources were used to interpret and organize the field but were not treated as equivalent to direct empirical confirmation of relationships (Peters et al., 2025; Arksey and O'Malley, 2005; Levac et al., 2010; Tricco et al., 2018; Rethlefsen et al., 2021).

Digital Compassion Fatigue and Digital Empathy were synthesized in parallel to identify areas of convergence, divergence, complementarity, and conceptual overlap, together with their relationships to adjacent constructs, including burnout, technostress, secondary traumatic stress, moral injury, resilience, and digital emotional labor. Particular attention was given to the influence of artificial intelligence and other emerging digital technologies on compassionate healthcare practice. The integrated findings informed the development of an evidence-informed Human-Centered Clinical Framework, which is presented and discussed in the Discussion section.

### 2.10 Risk of bias

Consistent with the Joanna Briggs Institute (JBI) methodology for scoping reviews, methodological quality appraisal and risk-of-bias assessment were not undertaken because the objective of this review was to map the breadth, characteristics, and conceptual development of the available evidence rather than to evaluate intervention effectiveness or estimate the risk of bias of individual studies (Peters et al., 2025; Arksey and O'Malley, 2005; Levac et al., 2010). This approach is also consistent with the PRISMA Extension for Scoping Reviews (PRISMA-ScR), which does not require critical appraisal unless assessment of methodological quality forms part of the review objectives. As the purpose of this review was evidence mapping and conceptual synthesis rather than evidence grading, no formal critical appraisal was performed.

## 3 Results

### 3.1 Search results

The literature search and study selection process are summarized in Figure 1. Following comprehensive literature searching, supplementary searching, duplicate removal, title and abstract screening, and full-text eligibility assessment, 41 evidence sources met the predefined eligibility criteria and were included in the final review synthesis. The search identified publications from 2016 to 2026, with a marked increase after 2020. Most studies originated from North America and Europe, and the majority focused on nursing, telehealth, electronic health records, or artificial intelligence. These evidence sources comprised concept analyses, scoping reviews, systematic reviews, meta-analyses, primary empirical studies, and theoretical or framework papers and constitute the evidence base summarized in Table 2.

In addition to the included evidence sources, the manuscript cites methodological guidance, measurement studies, theoretical literature, and other supporting references that informed the review methodology, conceptual interpretation, and development of the evidence-informed Human-Centered Clinical Framework. These references supported the narrative synthesis and discussion but were not included in the evidence mapping presented in Table 2.

### 3.2 Characteristics of the included evidence

The review synthesis was informed by 41 evidence sources representing diverse publication types, including concept analyses, primary empirical studies, qualitative and quantitative studies, scoping reviews, systematic reviews, meta-analyses, and theoretical or framework papers (Table 2). Collectively, these evidence sources spanned multiple healthcare professions, clinical settings, digital health technologies, and methodological approaches, reflecting the multidisciplinary and rapidly expanding evidence base on Digital Compassion Fatigue (DCF) and Digital Empathy.

Interpretation of the mapped evidence accounted for study type. Primary empirical studies were used to describe observed associations, experiences, and outcomes; systematic and scoping reviews were treated as secondary syntheses; and concept analyses, theoretical papers, commentaries, and framework studies were treated as conceptual evidence. Accordingly, statements about DCF as a distinct construct and about the proposed relationship between DCF and Digital Empathy should be read as conceptual propositions unless supported by primary empirical findings (Peters et al., 2025; Arksey and O'Malley, 2005; Levac et al., 2010; Tricco et al., 2018; Rethlefsen et al., 2021).

The included evidence demonstrated substantial differences in the maturity of the two focal concepts. Digital Compassion Fatigue remains an emerging construct and was explicitly addressed in only two concept analyses (Abou Hashish and Alnajjar, 2025; Byrne, 2025). No empirical study operationalized or measured DCF as a unified construct, and both concept analyses identified the absence of a validated instrument specifically designed to assess DCF.

In contrast, Digital Empathy is supported by a broader and more mature body of literature. The identified evidence included a nursing-specific concept analysis (Abou Hashish, 2025), the foundational pharmacy education commentary that first introduced the concept (Terry and Cain, 2016), and a comprehensive scoping review synthesizing 33 studies published between 2009 and 2026 across telehealth, telenursing, oncology, mental health, and AI-supported nursing contexts (Yildirim et al., 2026). Collectively, these studies conceptualized Digital Empathy as an adaptive competency that integrates interpersonal communication, technological proficiency, and patient-centered care within digitally mediated healthcare.

The evidence base also included an international eDelphi consensus study that developed a seven-domain competency framework for digital compassion in interprofessional healthcare practice (Wiljer et al., 2025). Adjacent systematic-review evidence also examined the contribution of artificial intelligence to electronic-health-record-related burnout among healthcare professionals (Sarraf and Ghasempour, 2025), reinforcing the importance of technology design and workload while remaining conceptually distinct from DCF.

Beyond these concept-specific publications, a substantial body of adjacent literature examined the psychological and relational consequences of digitally mediated healthcare. This included systematic reviews of electronic health record (EHR)-related burnout (Kroth et al., 2019; Hilliard et al., 2020; Alobayli et al., 2023; Wu et al., 2024), studies of technostress and digitally mediated work (Bail et al., 2023; Kasemy et al., 2022), and investigations of videoconference fatigue, including the development and cross-cultural validation of dedicated measurement instruments together with evidence demonstrating associations between videoconference fatigue and reduced provider empathy during telemedicine consultations (Fauville et al., 2021; Simbula et al., 2023; Myronuk, 2022).

### 3.3 Evolution of the field

The included evidence demonstrated a clear chronological progression in the development of research on digitally mediated compassion and empathy (Figure 2). The concept of Digital Empathy first appeared in a pharmacy education commentary in 2016 (Terry and Cain, 2016), several years before the rapid expansion of digitally mediated healthcare during the COVID-19 pandemic. Although the concept was initially introduced in educational settings, sustained conceptual and empirical research increased markedly from 2020 onward, paralleling the widespread adoption of telehealth, virtual care, and other digital healthcare technologies.

**Figure 2:**
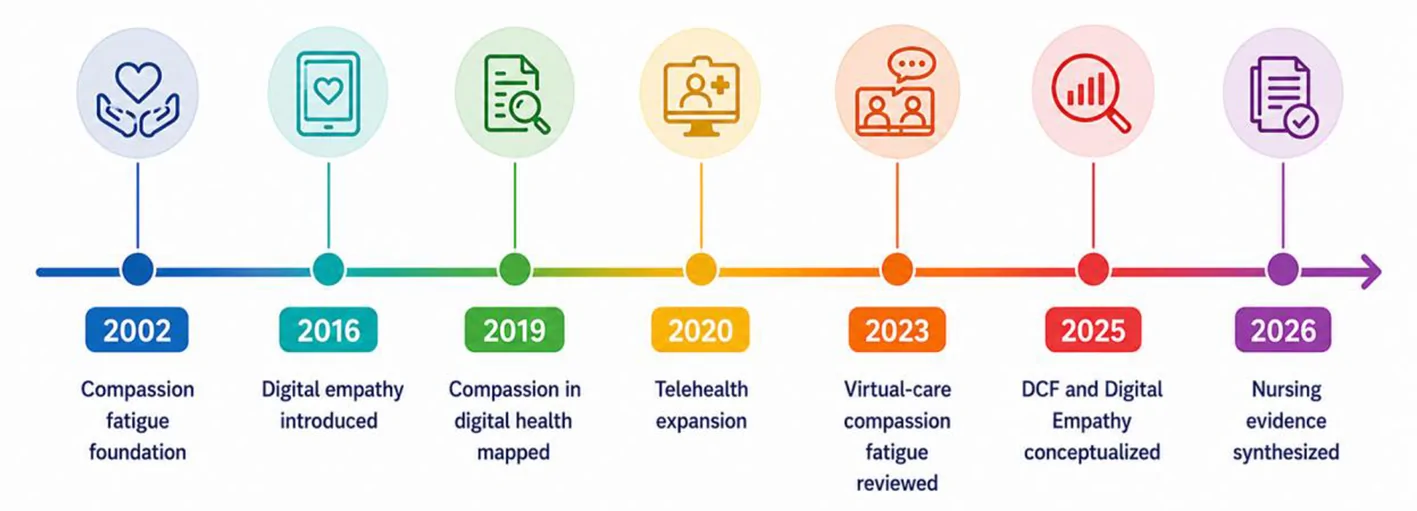
Chronological evolution of research on digital empathy and digital compassion fatigue. The figure depicts evidence development over time rather than causal or directional relationships between constructs.

Research focusing on compassion in digital healthcare also evolved progressively. Early scoping reviews examining compassion within digital health were published from 2019 (Wiljer et al., 2019; Kemp et al., 2020), followed by reviews addressing compassion fatigue in virtual care environments in 2023 (Crespo et al., 2023). In contrast, Digital Compassion Fatigue (DCF) emerged only recently as a distinct concept, with the first dedicated concept analyses published in 2025 (Abou Hashish and Alnajjar, 2025; Byrne, 2025), highlighting the early developmental stage of this field.

More recent contributions have expanded the evidence base substantially. A nursing-focused scoping review synthesized the literature on Digital Empathy across multiple healthcare contexts (Yildirim et al., 2026), while an international eDelphi consensus study developed a seven-domain competency framework for digital compassion in interprofessional healthcare practice (Wiljer et al., 2025). Together, these milestones illustrate the rapid maturation of research from early conceptual discussions toward structured evidence synthesis, competency development, and theoretical integration, reflecting growing recognition of the psychological and relational dimensions of digitally mediated healthcare.

### 3.4 Conceptualization of digital empathy

Across the included evidence, Digital Empathy was consistently conceptualized as an extension, rather than a replacement, of traditional empathy, referring to the ability to understand, communicate, and respond to patients' emotional and psychosocial needs through digitally mediated interactions while preserving compassionate, relational, and humanistic care (Abou Hashish, 2025; Terry and Cain, 2016; Yildirim et al., 2026). Collectively, the literature portrays Digital Empathy as an adaptive professional competency that integrates interpersonal communication, technological proficiency, and patient-centered care within digitally mediated healthcare environments.

The defining attributes consistently identified across the literature included authenticity, trust-building, effective digital communication, emotional engagement, adaptability, technological proficiency, and cultural sensitivity (Abou Hashish, 2025; Yildirim et al., 2026). Frequently reported antecedents comprised digital literacy, emotional intelligence, communication competence, and supportive technological and organizational infrastructures. Reported consequences included enhanced patient trust, stronger therapeutic relationships, greater patient satisfaction, improved perceived quality of care, and more effective clinician–patient interactions across digitally mediated healthcare settings (Abou Hashish, 2025; Yildirim et al., 2026).

Beyond communication skills alone, the broader evidence suggests that Digital Empathy represents a context-dependent capability that enables healthcare professionals to maintain compassionate, patient-centered relationships despite the relational challenges introduced by digital technologies. Table 3 summarizes the working definitions, theoretical foundations, defining attributes, and measurement status of the principal concepts identified in this review.

**Table 3:** Definitions, theoretical foundations, defining attributes, and measurement status of the principal concepts identified in the review.

Concept	Working definition	Defining attributes	Theoretical foundation	Measurement status	Distinguishing feature and overlap
Digital Empathy	The ability to understand, communicate, and respond to another person's emotional and psychosocial needs through digitally mediated interactions while preserving compassionate, relational, and humanistic care (Abou Hashish, 2025; Terry and Cain, 2016; Yildirim et al., 2026).	Authenticity; trust-building; effective digital communication; emotional engagement; adaptability; technological proficiency; cultural sensitivity	Extension of traditional empathy theory to technology-mediated healthcare interactions (Abou Hashish, 2025; Terry and Cain, 2016; Yildirim et al., 2026)	No validated healthcare-professional-specific instrument identified. Existing measures (e.g., CARE and DCES) provide partial proxies but do not comprehensively assess Digital Empathy in healthcare professionals (Mercer et al., 2004; Collins et al., 2024).	Digital-context adaptation of empathy. Overlaps with traditional empathy and digital compassion, but emphasizes relational understanding and response through technology rather than fatigue or distress. (Abou Hashish, 2025; Terry and Cain, 2016; Yildirim et al., 2026; Wiljer et al., 2025).
Digital Compassion Fatigue (DCF)	Sustained emotional exhaustion and empathic depletion experienced by healthcare professionals delivering continuous, digitally mediated care (Abou Hashish and Alnajjar, 2025; Byrne, 2025).	Emotional numbing; persistent post-engagement exhaustion; compassion withdrawal; cognitive overload; professional inefficacy; diminished therapeutic presence	Concept derived using Walker and Avant's concept analysis approach, informed by compassion fatigue and technostress theories (Abou Hashish and Alnajjar, 2025; Byrne, 2025; Stamm, 2012)	No validated DCF-specific measurement instrument has been developed (Abou Hashish and Alnajjar, 2025; Byrne, 2025).	Proposed technology-shaped compassion-related depletion. Overlaps with compassion fatigue, burnout, technostress, and secondary traumatic stress, but is theorized to require sustained digital demands alongside compassionate or empathic caregiving. (Abou Hashish and Alnajjar, 2025; Byrne, 2025; Maslach and Jackson, 1981; Figley, 2002; Peters, 2018; Sinclair et al., 2017).
Technostress	Psychological strain arising from difficulty adapting to the pace, complexity, and demands of information and communication technologies within professional practice (Bail et al., 2023; Wu et al., 2024; Kasemy et al., 2022).	Techno-overload; techno-complexity; techno-invasion; techno-insecurity; techno-uncertainty	Person–environment fit and stress-appraisal theories applied to technology use	Measured using general technostress creator/inhibitor scales; no Digital Empathy-specific instrument identified.	Technology-demand-specific strain that can occur without empathic caregiving. It may contribute to DCF but is not equivalent to DCF (Bail et al., 2023; Wu et al., 2024; Kasemy et al., 2022).
Burnout	A work-related syndrome characterized by emotional exhaustion, depersonalization or cynicism, and reduced personal accomplishment (Maslach and Jackson, 1981).	Emotional exhaustion; depersonalization/cynicism; reduced professional efficacy	Maslach's three-dimensional occupational burnout model (Maslach and Jackson, 1981)	Measured using the Maslach Burnout Inventory (MBI), the most widely validated instrument across healthcare professions.	Broad occupational syndrome not specific to digital care or exposure to others' suffering. Emotional exhaustion overlaps with DCF (Maslach and Jackson, 1981).
Compassion Fatigue	The cumulative emotional burden associated with caring for individuals experiencing suffering, encompassing elements of burnout and secondary traumatic stress (Figley, 2002; Peters, 2018; Sinclair et al., 2017).	Emotional exhaustion; reduced compassion satisfaction; secondary traumatic stress	Figley's compassion stress model (Figley, 2002)	Commonly measured using the Professional Quality of Life Scale (ProQOL) (Stamm, 2012).	Caregiving-related emotional cost that can occur in face-to-face or digital settings. DCF adds the proposed role of digitally mediated demands. (Figley, 2002; Peters, 2018; Sinclair et al., 2017; Crespo et al., 2023).
Secondary Traumatic Stress	Trauma-related psychological symptoms resulting from indirect exposure to patients' traumatic experiences through professional caregiving (Figley, 2002; Sinclair et al., 2017).	Intrusion; avoidance; hyperarousal	Indirect trauma and secondary traumatic stress theories	No digital healthcare-specific measurement approach was identified within the reviewed literature.	Trauma-symptom response to indirect exposure to others' trauma. It may coexist with DCF but does not require digital technology. (Figley, 2002; Sinclair et al., 2017).
Moral Injury	Psychological distress arising from actions, inactions, or circumstances that violate an individual's deeply held moral or ethical values.	Guilt; shame; betrayal; loss of trust	Originally developed in military trauma research and adapted to healthcare ethics	No measurement instrument specific to digitally mediated healthcare was identified in the reviewed literature.	Moral or ethical violation-related distress. It may overlap with DCF when digital systems constrain ethically congruent care, but the defining mechanism differs.
Digital Emotional Labor	The regulation and management of emotions required to maintain appropriate professional interactions during technology-mediated healthcare delivery.	Surface acting; deep acting; emotional regulation; emotional display management	Hochschild's emotional labor theory adapted to digitally mediated professional interactions	No validated instrument specific to digitally mediated healthcare was identified.	Emotion regulation during technology-mediated interaction. It may be an antecedent or correlate of DCF and a requirement for Digital Empathy, but is not itself empathy or fatigue. (Abou Hashish and Alnajjar, 2025; Abou Hashish, 2025; Luetke Lanfer et al., 2024; Byrne, 2025).

### 3.5 Conceptualization of digital compassion fatigue

Across the included evidence, Digital Compassion Fatigue (DCF) was conceptualized as a distinct form of emotional and empathic depletion associated with prolonged digitally mediated caregiving (Abou Hashish and Alnajjar, 2025; Byrne, 2025). Unlike traditional compassion fatigue, DCF specifically reflects the cumulative emotional demands of sustained screen-mediated clinical interactions, continuous digital connectivity, and technology-intensive healthcare practice. The two published concept analyses consistently identified six defining attributes: emotional numbing, persistent post-engagement exhaustion, compassion withdrawal, cognitive overload, professional inefficacy, and diminished therapeutic presence (Abou Hashish and Alnajjar, 2025; Byrne, 2025).

Reported antecedents clustered into three interrelated domains. Individual antecedents included prolonged digital caregiving, limited Digital Empathy competencies, insufficient digital communication skills, and ineffective coping strategies. Organizational antecedents comprised high digital caseloads, limited opportunities for supervision or emotional debriefing, inadequate organizational support, and rigid work schedules with insufficient recovery time. Technology-related antecedents included screen fatigue, poor platform usability, frequent connectivity disruptions, and increasing cognitive demands associated with digitally mediated healthcare systems (Abou Hashish and Alnajjar, 2025; Wu et al., 2024; Nadler, 2020; Byrne, 2025).

Because DCF remains an emerging concept, much of its theoretical foundation has been derived from the broader literature on compassion fatigue, burnout, and occupational stress. The reviewed evidence therefore included studies examining compassion satisfaction and coping strategies among nurses during the COVID-19 pandemic (Ruiz-Fernández et al., 2020), multinational investigations of compassion fatigue, burnout, and perceived stress (Xie et al., 2021), systematic reviews and meta-analyses of compassion fatigue and compassion satisfaction (Zhang et al., 2023), qualitative studies exploring nurses' lived experiences of compassion fatigue (Sorenson et al., 2016), and narrative reviews of compassion fatigue across healthcare settings (Sabery et al., 2017). Collectively, these studies provide the conceptual context supporting the emergence of DCF while highlighting the need for empirical validation and the development of dedicated measurement instruments (see Table 4 for the Comparison of digital empathy and digital compassion fatigue).

**Table 4:** Comparison of digital empathy and digital compassion fatigue.

Characteristic	Digital empathy	Digital compassion fatigue (DCF)
Conceptual nature	Adaptive professional capability that supports compassionate, patient-centered digital care	Proposed maladaptive response associated with prolonged digitally mediated caregiving
Definition	Ability to understand, communicate, and respond to patients' emotional and psychosocial needs through digitally mediated interactions while preserving compassionate care (Abou Hashish, 2025; Terry and Cain, 2016; Yildirim et al., 2026)	Sustained emotional exhaustion and empathic depletion associated with continuous digitally mediated caregiving (Abou Hashish and Alnajjar, 2025; Byrne, 2025)
Primary antecedents	Digital literacy; emotional intelligence; communication competence; supportive technological and organizational environments (Abou Hashish, 2025; Yildirim et al., 2026)	Proposed or reported antecedents/correlates include prolonged digital caregiving, high digital workload, inadequate organizational support, screen fatigue, poor platform usability, and limited digital communication resources (Abou Hashish and Alnajjar, 2025; Wu et al., 2024; Nadler, 2020; Byrne, 2025)
Defining attributes	Authenticity; trust-building; effective digital communication; emotional engagement; adaptability; technological proficiency; cultural sensitivity (Abou Hashish, 2025; Yildirim et al., 2026)	Emotional numbing; persistent post-engagement exhaustion; compassion withdrawal; cognitive overload; professional inefficacy; diminished therapeutic presence (Abou Hashish and Alnajjar, 2025; Byrne, 2025)
Typical consequences	Enhanced patient trust; stronger therapeutic relationships; improved patient satisfaction; higher perceived quality of care	Conceptually proposed or associated outcomes include burnout-related symptoms, compassion withdrawal, reduced therapeutic presence, diminished perceived quality of care, and possible workforce consequences
Theoretical foundation	Extension of traditional empathy theory to digitally mediated healthcare (Abou Hashish, 2025; Terry and Cain, 2016; Yildirim et al., 2026)	Compassion fatigue and technostress theories, conceptualized using Walker and Avant's concept analysis approach (Abou Hashish and Alnajjar, 2025; Byrne, 2025)
Measurement status	No validated healthcare-professional-specific instrument identified; existing measures (e.g., CARE and DCES) provide partial proxies (Mercer et al., 2004; Collins et al., 2024)	No validated DCF-specific measurement instrument currently available (Abou Hashish and Alnajjar, 2025; Byrne, 2025)
Evidence maturity	Developing but increasingly mature evidence base, including concept analyses, empirical studies, scoping reviews, and competency frameworks (Abou Hashish, 2025; Terry and Cain, 2016; Yildirim et al., 2026; Wiljer et al., 2025)	Early conceptual stage supported primarily by two concept analyses; no empirical studies measuring DCF as a unified construct (Abou Hashish and Alnajjar, 2025; Byrne, 2025)
Priority research needs	Cross-professional validation, cross-cultural studies, and development of healthcare-specific measurement instruments	Development and psychometric validation of DCF-specific instruments, empirical studies across healthcare settings, and longitudinal investigations of antecedents and outcomes

### 3.6 Shared antecedents, adjacent constructs, and the role of artificial intelligence

Figure 3 illustrates the distribution of evidence across digital healthcare technologies and related concepts identified in the review. Across the included evidence, Digital Compassion Fatigue (DCF) was consistently distinguished from, while partially overlapping with, related constructs including burnout (Maslach and Jackson, 1981), traditional compassion fatigue (Figley, 2002; Peters, 2018; Sinclair et al., 2017; Sabery et al., 2017), secondary traumatic stress, moral injury, and technostress (Bail et al., 2023; Wu et al., 2024; Kasemy et al., 2022). Although these constructs share common antecedents and psychological consequences, the literature consistently positioned DCF as a distinct phenomenon arising from the sustained emotional and cognitive demands of digitally mediated healthcare.

**Figure 3:**
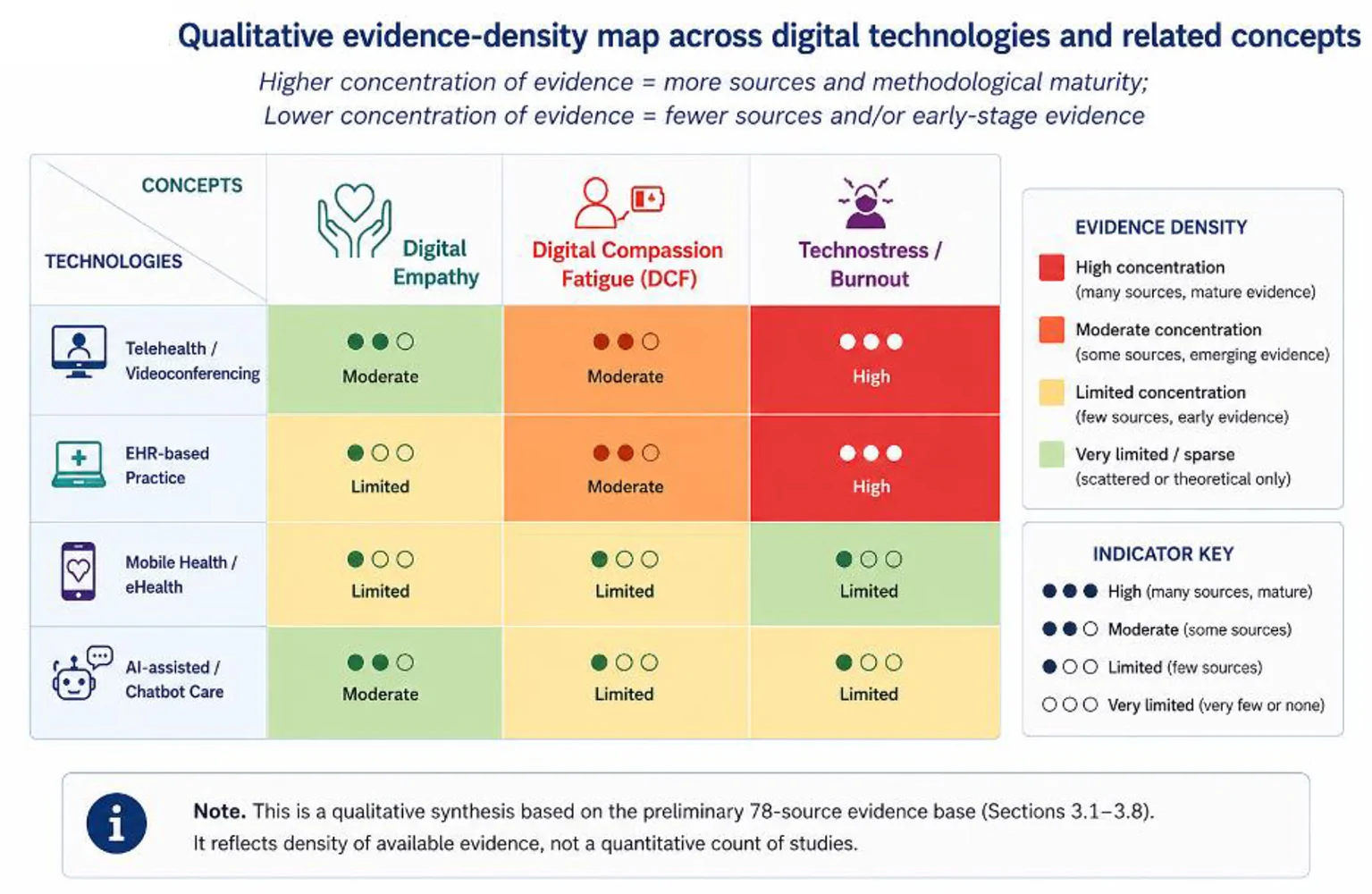
Distribution of the included evidence across digital healthcare technologies and related concepts.

Telehealth and videoconferencing were the most frequently represented digital healthcare contexts, followed by electronic health record (EHR)-supported practice, mobile and electronic health (mHealth/eHealth) applications, and artificial intelligence (AI)-assisted or chatbot-supported care (Yildirim et al., 2026; Tai-Seale et al., 2024; Garcia et al., 2024; Morrow et al., 2023; Girdwood et al., 2026). Evidence from an experimental crossover study demonstrated that patient-perceived physician empathy was significantly lower during EHR-supported consultations than during otherwise identical consultations conducted without computer use, suggesting that digital technologies may influence the quality of clinician–patient interactions when not implemented in a human-centered manner (Howcroft et al., 2025).

AI-related evidence represented the fastest-growing area of the literature and demonstrated both opportunities for supporting compassionate communication and risks related to depersonalization and reduced therapeutic presence. Several clinician-focused implementation studies reported that generative AI reduced documentation burden and produced patient messages perceived as highly empathetic (Tai-Seale et al., 2024; Garcia et al., 2024). Similarly, a recent systematic review and meta-analysis concluded that AI-generated responses were frequently rated as more empathetic than text-based responses written by healthcare professionals (Howcroft et al., 2025). Complementary experimental evidence further demonstrated that perceived empathy was influenced not only by response content but also by users' beliefs about whether the response originated from a human or an AI system (Muthukumar, 2025; Ruben et al., 2025).

Despite these promising findings, conceptual and qualitative studies consistently emphasized that AI cannot replace authentic human connection and cautioned that poorly implemented technologies may contribute to depersonalization, emotional distancing, and diminished therapeutic relationships (Luetke Lanfer et al., 2024; Morrow et al., 2023). Across the reviewed literature, empathy and compassion were frequently defined and operationalized inconsistently, particularly within AI research, highlighting an important conceptual limitation and reinforcing the need for an integrated, human-centered understanding of digitally mediated compassionate care.

### 3.7 Non-nursing professions, outcomes, and disciplinary distribution

Although nursing contributed a substantial proportion of concept-specific publications, related evidence also extends across health-professions education and other clinical disciplines. A scoping review of serious and digital games reported promising evidence for enhancing empathy among healthcare trainees, although the evidence remained limited (Dall et al., 2025). The broader cross-professional evidence includes physicians, psychologists, interdisciplinary healthcare professionals, health-professions learners, and patient evaluations of clinician or AI communication.

Evidence involving other healthcare professions was comparatively limited and rarely used the terms Digital Compassion Fatigue or Digital Empathy explicitly. Among psychologists, studies reported increased compassion fatigue, higher caseload intensity, and challenges associated with telehealth delivery during the COVID-19 pandemic (Kercher et al., 2024). Additional evidence identified elevated levels of personal, work-related, and client-related burnout among psychologists engaged in teleworking (Serrão et al., 2022). In contrast, physician-focused research was more extensive, particularly regarding electronic health record (EHR)-related burnout and the use of artificial intelligence to support clinical communication, although these studies generally examined burnout, workload, or communication quality rather than Digital Compassion Fatigue or Digital Empathy (Mercer et al., 2004; Gilbert, 2009; Luetke Lanfer et al., 2024; Duffy et al., 2023; Maslach and Jackson, 1981; Tai-Seale et al., 2024; Garcia et al., 2024; Howcroft et al., 2025; Muthukumar, 2025; Topol, 2019).

Evidence concerning videoconference fatigue showed mixed findings. Some studies reported increased cognitive load, reduced non-verbal communication, interface-related stress, and greater fatigue among healthcare professionals using videoconferencing technologies (Fauville et al., 2021; Simbula et al., 2023; Khan et al., 2025; Khairat et al., 2025). In contrast, a longitudinal study of mental health professionals found no association between videoconferencing and fatigue and reported declining burnout over time despite continued virtual practice (Steidtmann et al., 2024). These findings suggest that the psychological effects of digitally mediated communication may vary across professional groups, clinical contexts, and patterns of technology use.

Across the reviewed evidence, reported outcomes clustered into three broad domains: professional outcomes, including burnout, professional inefficacy, turnover intention, and reduced wellbeing; relational outcomes, including patient trust, therapeutic alliance, patient satisfaction, and communication quality; and organizational outcomes, including quality of care, workforce retention, and the safe and effective implementation of digital healthcare technologies. These outcome domains informed the development of the evidence-informed Human-Centered Clinical Framework presented in the Discussion.

### 3.8 Evidence and measurement gaps

The included evidence demonstrated considerable heterogeneity in the measurement approaches used to investigate empathy, compassion fatigue, burnout, and related psychological constructs in digitally mediated healthcare. Although several validated instruments were identified, none was specifically developed to measure Digital Compassion Fatigue (DCF) as a distinct construct (Abou Hashish and Alnajjar, 2025; Byrne, 2025). Instead, researchers relied on established measures assessing related domains such as compassion satisfaction, burnout, empathy, videoconference fatigue, telehealth usability, and digital communication.

The most frequently reported instruments included the Professional Quality of Life Scale (ProQOL) (Stamm, 2012), the Maslach Burnout Inventory (MBI) (Maslach and Jackson, 1981), the Zoom Exhaustion and Fatigue Scale (ZEFS) (Fauville et al., 2021; Simbula et al., 2023), the Digital Communication Empathy Scale (DCES) (Collins et al., 2024), the Consultation and Relational Empathy (CARE) Measure (Mercer et al., 2004), the Telehealth Usability Questionnaire (TUQ) (Parmanto et al., 2016), and the Copenhagen Burnout Inventory (CBI) (Serrão et al., 2022). While these instruments provide valuable insights into individual aspects of digitally mediated healthcare, none comprehensively captures the multidimensional characteristics of Digital Compassion Fatigue or simultaneously evaluates Digital Compassion Fatigue and Digital Empathy within a unified measurement framework.

The review also identified growing interest in technology-supported interventions designed to improve clinician wellbeing. A recent systematic review and meta-analysis reported promising findings for mobile application-based interventions targeting burnout, compassion fatigue, and compassion satisfaction among healthcare professionals (Knop et al., 2024). Nevertheless, no study simultaneously examined Digital Compassion Fatigue and Digital Empathy within the same analytical framework, highlighting an important conceptual and methodological gap in the current literature. The characteristics, applications, and limitations of the principal measurement instruments identified in the included evidence are summarized in Table 5.

**Table 5:** Measurement instruments identified in the included evidence.

Instrument	Primary construct(s) measured	Digital-specific	Healthcare validation	Relevance and limitations for digital compassion fatigue and digital empathy
Consultation and Relational Empathy (CARE) Measure (Mercer et al., 2004)	Patient-perceived clinician empathy and therapeutic relationship	No	Extensively validated in face-to-face healthcare settings	Measures clinician empathy from the patient's perspective but has not been validated for video consultations, asynchronous communication, or digitally mediated clinical encounters.
Digital Communication Empathy Scale (DCES) (Walker and Avant, 2011)	Empathy expressed through digital communication	Yes	Not validated in healthcare-professional clinical samples	Assesses empathy during digital communication but was developed in general population settings and does not evaluate Digital Compassion Fatigue or healthcare-specific Digital Empathy.
Zoom Exhaustion and Fatigue Scale (ZEFS) (Fauville et al., 2021; Simbula et al., 2023)	General, visual, emotional, motivational, and social videoconference fatigue	Yes	Applied in general and healthcare videoconferencing contexts	Measures videoconference-related fatigue but does not assess compassion, empathic engagement, or technology-mediated caregiving.
Professional Quality of Life Scale (ProQOL) (Stamm, 2012)	Compassion satisfaction, burnout, and secondary traumatic stress	No	Extensively validated across healthcare and helping professions	Widely used to evaluate professional quality of life but does not distinguish digitally mediated from face-to-face caregiving or capture Digital Compassion Fatigue-specific characteristics.
Maslach Burnout Inventory (MBI) (Maslach and Jackson, 1981)	Emotional exhaustion, depersonalization, and reduced personal accomplishment	No	Extensively validated across healthcare professions	Measures occupational burnout but is not designed to assess technology-related emotional demands or Digital Compassion Fatigue.
Telehealth Usability Questionnaire (TUQ) (Parmanto et al., 2016)	Telehealth usability, interface quality, interaction quality, reliability, usefulness, and satisfaction	Yes	Validated in telehealth settings	Assesses telehealth system usability rather than clinicians' emotional, relational, or empathic experiences during digitally mediated care.
Copenhagen Burnout Inventory (CBI) (Serrão et al., 2022)	Personal, work-related, and client-related burnout	No	Validated across healthcare and helping professions	Measures burnout across multiple domains but does not specifically evaluate Digital Compassion Fatigue or digitally mediated caregiving experiences.
Digital Compassion Fatigue-specific instrument (Abou Hashish and Alnajjar, 2025; Byrne, 2025)	Digital Compassion Fatigue	—	Not available	No validated instrument specifically designed to measure Digital Compassion Fatigue was identified, representing the principal measurement gap highlighted by this review.

## 4 Discussion

### 4.1 Digital healthcare creates both opportunities and risks for compassionate care

The mapped evidence suggests that digitally mediated healthcare may create opportunities to support compassionate, patient-centered care while also being associated with emotional exhaustion and compassion-related burden among healthcare professionals. Digital Empathy and DCF are therefore interpreted here as related but non-oppositional responses within the same digital clinical context. This interpretation is evidence-informed but not causally established because much of the underlying literature is cross-sectional, conceptual, or secondary (Abou Hashish, 2025; Luetke Lanfer et al., 2024; Yildirim et al., 2026; Kroth et al., 2019; Hilliard et al., 2020; Alobayli et al., 2023; Wu et al., 2024; Kasemy et al., 2022; Tai-Seale et al., 2024; Garcia et al., 2024; Morrow et al., 2023; Wiljer et al., 2025; Ali et al., 2022; Crespo et al., 2023).

The synthesis supports four interconnected interpretations. First, compassion can be adapted rather than necessarily diminished within digitally mediated healthcare. Second, Digital Empathy and DCF are better treated as distinct, potentially co-occurring responses than as opposite ends of a single continuum. Third, individual, technological, and organizational conditions may shape these responses, although the direction and magnitude of these relationships require empirical testing. Fourth, human-centered digital transformation should evaluate relational quality alongside technological performance. These interpretations provide the conceptual basis for the evidence-informed Human-Centered Clinical model proposed in this review.

### 4.2 Compassion is evolving rather than disappearing

A longstanding concern surrounding healthcare digitalization is that increasing reliance on technology may weaken the humanistic foundations of clinical practice. The evidence synthesized in this review does not support this assumption. Instead, Digital Empathy was consistently described as an adaptive extension of traditional empathy that enables healthcare professionals to preserve meaningful therapeutic relationships across telehealth, asynchronous communication, and other digitally mediated care environments (Abou Hashish, 2025; Terry and Cain, 2016; Yildirim et al., 2026).

The nursing-focused scoping review by (Yildirim et al. 2026) concluded that Digital Empathy represents a context-dependent evolution of traditional empathy rather than a fundamentally different construct. Similar conclusions emerged from studies evaluating generative AI-supported clinical communication, where AI-generated message drafts reduced documentation burden while supporting clinicians in producing responses perceived as empathetic, provided that clinicians retained responsibility for reviewing, personalizing, and contextualizing the final communication (Tai-Seale et al., 2024; Garcia et al., 2024). These findings are further supported by the broader literature, which argues that the greatest value of artificial intelligence lies in augmenting rather than replacing human compassion by reducing administrative burden and allowing clinicians to devote greater attention to patient relationships (Everett et al., 2026).

At the same time, the evidence indicates that this adaptive evolution is not inevitable. The quality of compassionate digital care appears to depend on the interaction between technology design, digital competencies, organizational support, workload, and opportunities for meaningful clinician–patient engagement. Digital Compassion Fatigue may therefore represent a maladaptive response associated with digitally mediated care when these enabling conditions are insufficient. This interpretation may help explain why adaptive and maladaptive responses coexist across the current evidence base and provides a conceptual basis for the Human-Centered Clinical Framework developed in this review.

### 4.3 Digital empathy and digital compassion fatigue are complementary rather than opposing responses

A central interpretive proposition arising from this review is that Digital Empathy and Digital Compassion Fatigue (DCF) should not be viewed as opposite ends of a single continuum. The available literature is more consistent with treating them as distinct and potentially co-occurring responses within the same digitally mediated healthcare environment. Healthcare professionals may maintain empathic digital practice while also experiencing emotional exhaustion, but this co-occurrence has not yet been tested directly using validated measures of both constructs (Abou Hashish and Alnajjar, 2025; Byrne, 2025; Gustafsson and Hemberg, 2022; Labrague, 2021).

Conversely, the broader evidence suggests that supportive organizational cultures, psychologically safe work environments, manageable digital workloads, well-designed technologies, and appropriate education may support empathic digital practice and potentially reduce conditions associated with compassion-related depletion (World Health Organization, 2021; Gilbert, 2014). Evidence from the electronic health record (EHR) literature further reinforces this interpretation by demonstrating that clinician burnout is associated with modifiable workload, workflow, and system-design factors rather than with technology use alone (Kroth et al., 2019; Hilliard et al., 2020; Alobayli et al., 2023; Wu et al., 2024). These findings suggest that digital technologies are not inherently protective or harmful; their psychological impact may depend on how they are implemented, integrated into clinical workflows, and supported within healthcare organizations.

This interpretation challenges the assumption that increasing Digital Empathy necessarily protects against Digital Compassion Fatigue or that the presence of one excludes the other. Instead, both responses may coexist within the same clinician, healthcare team, or period of practice, reflecting different adaptations to shared individual, technological, and organizational conditions. This conceptual positioning distinguishes the present review from previous work. The nursing-focused scoping review synthesized the evidence on Digital Empathy without examining its potential emotional costs (Yildirim et al., 2026), whereas the international eDelphi study identified competencies required for digital compassion but did not consider how these competencies may be affected by prolonged digital workload or compassion-related depletion (Wiljer et al., 2025).

Accordingly, this review proposes that Digital Empathy and Digital Compassion Fatigue should be conceptualized as interrelated yet distinct outcomes arising from common antecedents rather than as opposing constructs. This interpretation provides the theoretical foundation for the evidence-informed Human-Centered Clinical Framework, which integrates adaptive and maladaptive pathways within digitally mediated healthcare (Figure 4).

**Figure 4:**
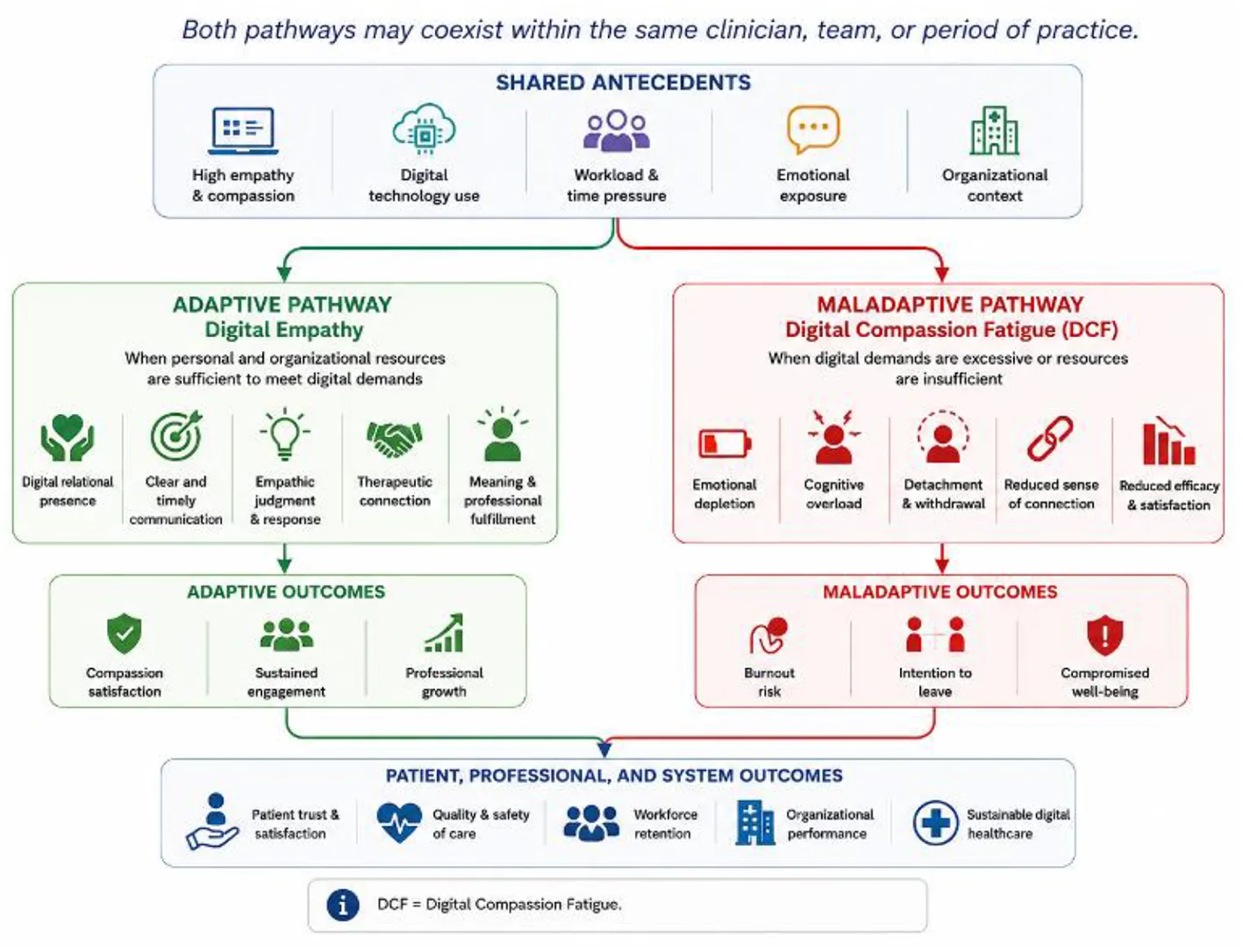
Conceptual pathways linking digital empathy and digital compassion fatigue. Digital Empathy and Digital Compassion Fatigue are represented as related, potentially co-occurring adaptive and maladaptive responses arising within shared digital contexts. The pathways are conceptual, potentially reciprocal, and non-causal.

### 4.4 Adaptive resources and organizational support shape digital healthcare outcomes

If Digital Empathy and Digital Compassion Fatigue (DCF) represent complementary rather than opposing responses to digitally mediated healthcare, the key question becomes which factors influence whether adaptive or maladaptive outcomes predominate. The evidence synthesized in this review indicates that this balance is shaped by interacting individual and organizational resources rather than by technology alone.

At the individual level, digital competence, emotional intelligence, resilience, professional identity, and reflective practice were consistently identified as protective factors across the Digital Empathy, technostress, and digital wellbeing literature (Abou Hashish, 2025; Bail et al., 2023; Kasemy et al., 2022; Labrague, 2021). These competencies appear to strengthen clinicians' ability to maintain compassionate relationships while adapting to increasingly technology-intensive healthcare environments. Although not directly examined within the reviewed digital healthcare literature, self-compassion has been consistently associated with reduced burnout and greater psychological resilience in the broader compassion science literature (Neff, 2003, International Organization for Standardization (ISO) 9241–210). Its potential role as a protective factor against Digital Compassion Fatigue therefore represents an important direction for future research.

Digital literacy and digital competence warrant particular attention because clinicians and patients do not enter digital care with equivalent skills, confidence, access, or familiarity. Limited competence may increase cognitive effort, workflow disruption, uncertainty, and technostress for professionals, while limited patient digital literacy may require additional explanation and relational work from clinicians. These inequalities may therefore amplify digital workload and shape both perceived empathy and professional wellbeing. The present evidence does not establish digital competence as a causal protective factor against DCF, but it identifies competence as a plausible, modifiable resource for future testing (Abou Hashish, 2025; Alotaibi et al., 2025; Bail et al., 2023; Yildirim et al., 2026; Kasemy et al., 2022; Wiljer et al., 2025).

At the organizational level, the evidence consistently emphasized the importance of leadership support, psychological safety, adequate staffing, user-centered technology design, digital training, and opportunities for recovery between digitally mediated clinical encounters (Abou Hashish and Alnajjar, 2025; Bail et al., 2023; Mercer et al., 2004; Gilbert, 2009; Luetke Lanfer et al., 2024; Duffy et al., 2023). Evidence from studies of electronic health records further demonstrated that clinician burnout is strongly influenced by modifiable workflow and system-design characteristics rather than technology use itself (Kroth et al., 2019; Hilliard et al., 2020; Alobayli et al., 2023; Wu et al., 2024). Together, these findings suggest that sustainable digitally mediated healthcare depends on the interaction between individual adaptive capabilities and supportive organizational environments. Neither level alone appears sufficient to protect healthcare professionals from compassion-related depletion or to sustain compassionate digital practice.

These interacting individual, organizational, and technological influences constitute the principal mechanism represented within the evidence-informed Human-Centered Clinical Framework proposed in this review.

### 4.5 Human-centered digital transformation requires relational quality as a core outcome

The findings of this review suggest that digital transformation should be evaluated not only by improvements in efficiency and productivity but also by its capacity to preserve compassionate, patient-centered care. Current digital health initiatives frequently emphasize operational indicators, including documentation efficiency, consultation volume, and workflow performance, whereas relational outcomes such as perceived empathy, therapeutic presence, patient trust, and communication quality receive comparatively less attention.

The evidence relating to generative artificial intelligence illustrates this challenge. Studies included in this review reported that AI-assisted message drafting reduced clinicians' documentation burden and, in some settings, generated responses perceived as highly empathetic (Tai-Seale et al., 2024; Garcia et al., 2024). However, conceptual and qualitative studies also cautioned that AI may contribute to depersonalization or emotional distancing when implemented without attention to relational design and human oversight (Luetke Lanfer et al., 2024; Morrow et al., 2023). Collectively, these findings indicate that the effects of AI depend less on the technology itself than on how it is integrated into clinical workflows and supported by organizational policies and professional judgment.

These observations have important implications for digital transformation in healthcare. Technology procurement, workflow redesign, digital governance, and AI implementation should evaluate relational quality alongside technical performance, efficiency, and usability. This interpretation aligns with the World Health Organization's guidance on ethical artificial intelligence in health, which emphasizes that human values and ethical principles should be embedded throughout the design and implementation of digital health systems (Deriglazov et al., 2025). It is also consistent with established human-centered design principles, which advocate integrating user wellbeing, safety, and meaningful human interaction as fundamental design requirements rather than secondary implementation outcomes (Biggs et al., 2017).

Accordingly, the evidence synthesized in this review supports a model of human-centered digital transformation in which technological innovation and compassionate healthcare are regarded as mutually reinforcing rather than competing priorities. This interpretation provides the conceptual foundation for the evidence-informed Human-Centered Clinical Framework presented in the following section.

### 4.6 Toward an evidence-informed human-centered clinical framework

Building on the evidence synthesized in this review, we propose an evidence-informed Human-Centered Clinical conceptual framework that integrates the principal concepts, antecedents, and outcomes identified across the literature (Figure 5). The framework is a testable conceptual synthesis derived from the current evidence rather than an empirically validated clinical model and therefore provides a foundation for future theory development, empirical testing, and intervention design.

**Figure 5:**
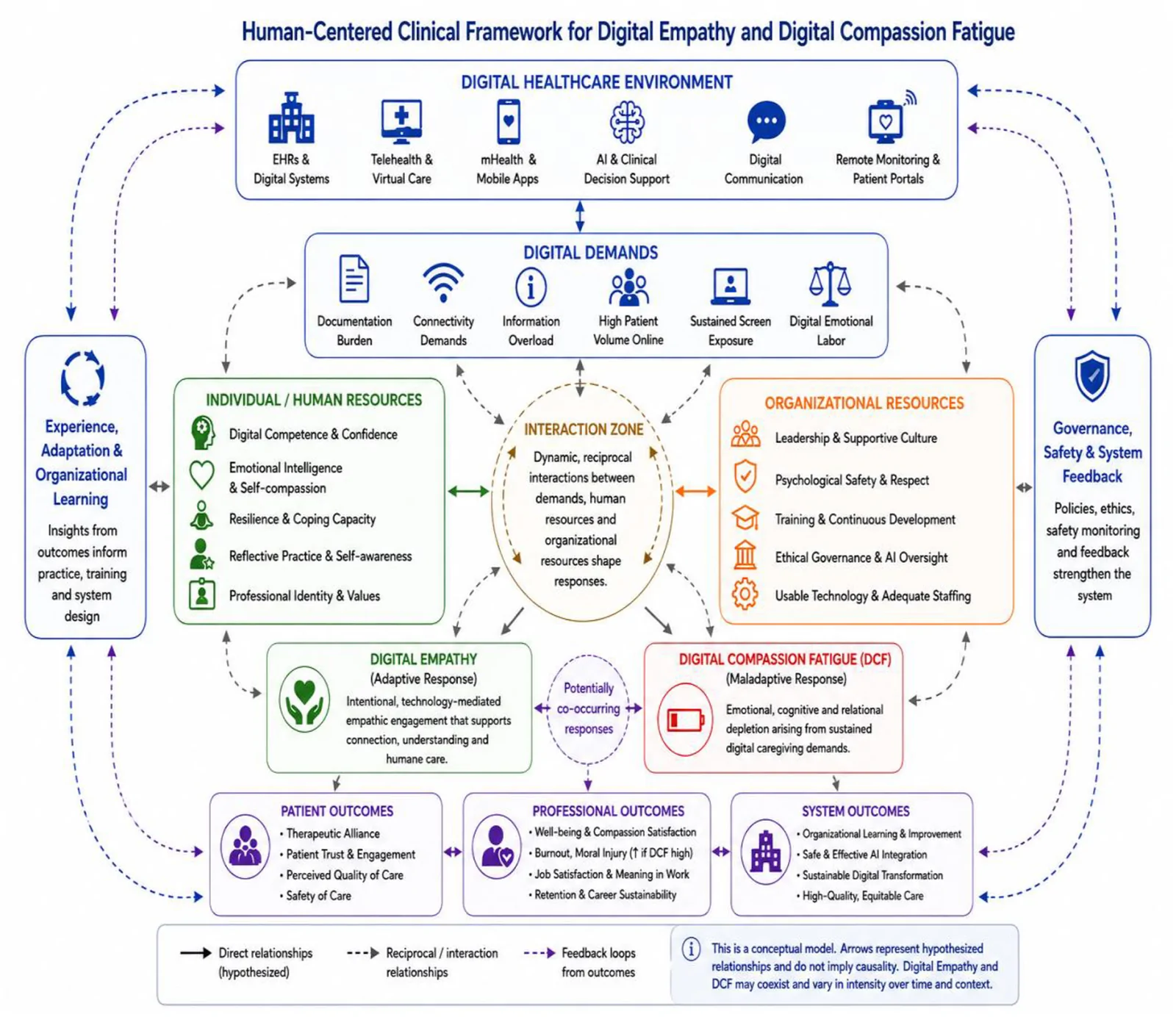
Evidence-informed human-centered clinical conceptual model. Arrows represent hypothesized, potentially reciprocal relationships derived from the scoping synthesis and should not be interpreted as established causal pathways.

Framework development followed an explicit evidence-to-domain mapping process. Digital demands were derived from recurring findings on EHR workload, continuous connectivity, videoconference fatigue, information overload, and AI-mediated work (Kroth et al., 2019; Hilliard et al., 2020; Alobayli et al., 2023; Wu et al., 2024; Kasemy et al., 2022; Nadler, 2020; Fauville et al., 2021; Simbula et al., 2023; Myronuk, 2022; Tai-Seale et al., 2024; Garcia et al., 2024; Morrow et al., 2023). Individual resources were mapped from Digital Empathy, technostress, resilience, and communication literature (Abou Hashish, 2025; Bail et al., 2023; Yildirim et al., 2026; Kasemy et al., 2022; Labrague, 2021). Organizational resources were mapped from evidence concerning leadership support, staffing, workflow, psychological safety, user-centered design, and governance (Kroth et al., 2019; Hilliard et al., 2020; Alobayli et al., 2023; Wu et al., 2024; Wiljer et al., 2025; Gunawan, 2026; World Health Organization, 2021). Patient and professional outcomes were drawn from reported relational, wellbeing, and workforce outcomes across the included studies. The adaptive and maladaptive pathways represent the author's integrative synthesis of these mapped domains; they have not been tested as directional or causal pathways (Abou Hashish and Alnajjar, 2025; Abou Hashish, 2025; Bail et al., 2023; Yildirim et al., 2026; Kroth et al., 2019; Hilliard et al., 2020; Alobayli et al., 2023; Wu et al., 2024; Kasemy et al., 2022; Nadler, 2020; Fauville et al., 2021; Simbula et al., 2023; Myronuk, 2022; Byrne, 2025; Labrague, 2021; Tai-Seale et al., 2024; Garcia et al., 2024; Morrow et al., 2023; Wiljer et al., 2025; Gunawan, 2026; World Health Organization, 2021).

The framework should be interpreted as dynamic and reciprocal rather than as a linear top-to-bottom sequence. Digital demands, adaptive resources, organizational conditions, relational experiences, and professional outcomes may influence one another over time. For example, fatigue may reduce therapeutic presence, while difficult relational encounters may increase emotional labor; supportive workflow redesign may reduce burden, while accumulating burden may alter how technology is used. Arrows in the model therefore denote hypothesized relationships for future testing rather than established causal effects (Kroth et al., 2019; Hilliard et al., 2020; Alobayli et al., 2023; Wu et al., 2024; Kasemy et al., 2022; Maslach and Jackson, 1981; Labrague, 2021; Gunawan, 2026; Proctor et al., 2011).

The framework complements, rather than replaces, existing models of digital compassion. For example, the interprofessional digital compassion competency framework developed through the international eDelphi study focuses on the competencies required to deliver compassionate digital care at the point of practice (Wiljer et al., 2025). In contrast, the framework proposed in the present review adopts a broader systems perspective by integrating the individual, technological, organizational, and environmental conditions that shape whether digitally mediated healthcare promotes Digital Empathy or contributes to Digital Compassion Fatigue (DCF) over time.

As illustrated in Figure 5, the framework comprises five interrelated layers. The digital healthcare environment forms the contextual foundation and includes technologies such as artificial intelligence, telehealth, electronic health records, remote monitoring systems, patient portals, and digital communication platforms. These technologies generate digital demands, including documentation burden, continuous connectivity, virtual communication, information overload, artificial intelligence oversight, and digital emotional labor. The impact of these demands is moderated by individual adaptive resources, including digital competence, emotional intelligence, resilience, self-awareness, professional identity, reflective practice, and psychological flexibility, together with organizational resources, such as supportive leadership, psychological safety, adequate staffing, education and training, user-centered technology design, and ethical governance of artificial intelligence.

The interaction among these contextual, technological, individual, and organizational factors determines whether healthcare professionals are more likely to experience the adaptive pathway, characterized by Digital Empathy and compassionate digital practice, or the maladaptive pathway, characterized by Digital Compassion Fatigue. Rather than representing opposite ends of a single continuum, these pathways are conceptualized as distinct but interrelated responses arising from shared antecedents within digitally mediated healthcare environments.

The framework further proposes that these adaptive and maladaptive pathways extend beyond individual clinicians to influence patient-level outcomes, including therapeutic relationships, patient trust, communication quality, and perceived quality of care, as well as system-level outcomes, including clinician wellbeing, workforce retention, organizational resilience, safe artificial intelligence implementation, and the long-term sustainability of digital transformation in healthcare. The proposed framework is theoretically informed by stress and coping models of technostress (Proctor et al., 2011), occupational burnout theory (Maslach and Jackson, 1981), and the developing conceptual literature on Digital Compassion Fatigue and Digital Empathy (Abou Hashish and Alnajjar, 2025; Abou Hashish, 2025; Byrne, 2025), while incorporating evidence from studies of electronic health record design (Kroth et al., 2019; Hilliard et al., 2020; Alobayli et al., 2023; Wu et al., 2024), videoconference fatigue (Fauville et al., 2021; Simbula et al., 2023; Myronuk, 2022), telehealth, and artificial intelligence. Because the framework is derived from evidence synthesis rather than empirical testing, it should be regarded as a testable conceptual model. Future research should examine the proposed relationships, validate the framework across healthcare professions and settings, and develop standardized instruments capable of measuring both Digital Compassion Fatigue and Digital Empathy within a unified conceptual framework.

### 4.7 Clinical significance

The proposed framework has potential practical implications at multiple levels of healthcare delivery. For individual healthcare professionals, it offers a structured way to consider how persistent digital fatigue may be associated with the interaction between digital demands and available personal and organizational resources rather than with individual coping alone. Digital Compassion Fatigue should therefore be regarded as a potential occupational response requiring empirical validation. The framework may help identify modifiable conditions for future intervention testing.

At the organizational level, the framework may help identify whether compassion-related challenges are associated primarily with limitations in individual competencies, organizational support, technology design, or interactions among these factors. This approach may inform the selection and future evaluation of interventions that address underlying conditions rather than relying solely on additional training or technological solutions.

At the health-system level, the framework conceptually links clinician wellbeing with broader organizational outcomes, including workforce retention, patient trust, quality of care, and the safe implementation of artificial intelligence. Supporting Digital Empathy and addressing conditions potentially associated with Digital Compassion Fatigue may therefore represent important components of sustainable digital transformation rather than isolated workforce wellbeing initiatives.

### 4.8 Digital compassion as an organizational capability

Although the preceding discussion emphasizes the experiences of individual healthcare professionals, the evidence synthesized in this review demonstrates that many antecedents of Digital Compassion Fatigue and Digital Empathy—including electronic health record design, workload distribution, leadership practices, education, organizational culture, and artificial intelligence governance—are fundamentally organizational rather than individual in nature. This observation supports the proposition that digital compassion may also be conceptualized as an organizational capability extending beyond individual professional competence.

Implementation science provides a useful perspective for interpreting these findings. Successful implementation of digital health technologies depends not only on the technical performance of the intervention but also on organizational factors such as acceptability, feasibility, fidelity, and long-term sustainability (Abou Hashish and Alnajjar, 2025; Abou Hashish, 2025). Consequently, leadership, psychologically safe work environments, workflow design, user-centered technology development, and ethical governance of artificial intelligence should be regarded as essential determinants of compassionate digital care rather than contextual considerations.

Evidence synthesized in this review further suggests that identical digital technologies may produce substantially different clinician experiences depending on how they are implemented. A randomized study of clinician–AI collaborative diagnostic workflows demonstrated that alternative approaches to integrating the same artificial intelligence system resulted in different clinician experiences and outcomes, highlighting the importance of workflow design and implementation strategy (Gunawan, 2026). These findings reinforce the proposition that organizational conditions influence whether individual competencies are sufficient to sustain Digital Empathy or whether clinicians become increasingly vulnerable to Digital Compassion Fatigue.

### 4.9 Implications for practice, education, leadership, technology design, policy, and research

What this review adds. The review moves beyond profession-specific mapping by integrating Digital Empathy, DCF, and adjacent digital-burden constructs across healthcare professions and technologies. It clarifies where evidence is empirical vs. conceptual, identifies the absence of a validated DCF measure, distinguishes perceived AI empathy from human empathic capacity and clinical outcomes, and proposes a testable cross-level model linking digital demands, resources, relational processes, and outcomes. These additions provide hypotheses and measurement priorities rather than validated clinical pathways (Abou Hashish and Alnajjar, 2025; Abou Hashish, 2025; Yildirim et al., 2026; Byrne, 2025; Wiljer et al., 2019; Kemp et al., 2020; Wiljer et al., 2025; Ali et al., 2022; Crespo et al., 2023).

The evidence synthesized in this review has implications for multiple stakeholder groups (Table 6). For clinicians, Digital Compassion Fatigue should be recognized as an occupational indicator of digital workload rather than solely an individual coping challenge. Healthcare educators should integrate Digital Empathy, artificial intelligence communication, digital professionalism, and emotional self-regulation into health-professions curricula alongside conventional communication skills (Abou Hashish, 2025; Yildirim et al., 2026; Gunawan, 2026; Pepito et al., 2026). Healthcare leaders should prioritize psychologically safe digital work environments while addressing modifiable workload and electronic health record design factors consistently associated with clinician burnout (Kroth et al., 2019; Hilliard et al., 2020; Alobayli et al., 2023; Wu et al., 2024).

**Table 6:** Implications of the Review for Key Stakeholders.

Stakeholder	Key recommendation	Supporting evidence
Healthcare professionals	Consider Digital Compassion Fatigue as a potential occupational indicator of digital workload and support Digital Empathy through reflective practice, emotional self-regulation, and appropriate recovery strategies.	Abou Hashish and Alnajjar, 2025; Abou Hashish, 2025; Bouabida et al., 2022
Educators	Integrate Digital Empathy, artificial intelligence communication, digital professionalism, emotional self-regulation, and compassionate digital care into health-professions education.	Abou Hashish, 2025; Yildirim et al., 2026; Gunawan, 2026; Pepito et al., 2026
Healthcare leaders and managers	Develop psychologically safe digital workplaces, optimize staffing and workflow, and address modifiable electronic health record and workload factors associated with clinician wellbeing.	Abou Hashish and Alnajjar, 2025; Bail et al., 2023; Kroth et al., 2019; Hilliard et al., 2020; Alobayli et al., 2023; Wu et al., 2024
Technology developers & industry partners	Apply human-centered design principles and design artificial intelligence to support rather than substitute for compassionate clinician–patient interactions.	Luetke Lanfer et al., 2024; Tai-Seale et al., 2024; Garcia et al., 2024; Morrow et al., 2023; Girdwood et al., 2026
Policymakers	Incorporate relational-quality indicators into digital transformation strategies and strengthen ethical governance of artificial intelligence in healthcare.	Deriglazov et al., 2025; Biggs et al., 2017
Researchers	Develop validated Digital Compassion Fatigue instruments, conduct cross-professional and longitudinal studies, and empirically evaluate the proposed Human-Centered Clinical Framework.	Abou Hashish and Alnajjar, 2025; Abou Hashish, 2025; Yildirim et al., 2026; Byrne, 2025; Wiljer et al., 2025

Technology developers should adopt human-centered design principles to ensure that artificial intelligence and other digital technologies strengthen rather than replace therapeutic relationships (Luetke Lanfer et al., 2024; Tai-Seale et al., 2024; Garcia et al., 2024; Morrow et al., 2023). Policymakers should incorporate indicators of relational quality alongside conventional digital transformation metrics and promote ethical governance of artificial intelligence throughout digital health implementation. Finally, researchers should prioritize the development and validation of Digital Compassion Fatigue measurement instruments, cross-professional and cross-cultural studies, longitudinal investigations, and empirical testing of the proposed Human-Centered Clinical Framework. These recommendations are summarized in Table 6.

### 4.10 Future research agenda

The findings of this review identify several priorities for advancing research on Digital Compassion Fatigue (DCF) and Digital Empathy. Conceptually, future research should establish consensus definitions and further clarify the boundaries between DCF, burnout, technostress, traditional compassion fatigue, and related constructs. Particular attention should be given to refining the theoretical relationships among these concepts within digitally mediated healthcare.

Measurement represents the highest research priority. The review identified no validated instrument specifically designed to measure DCF, highlighting an important methodological gap. Future studies should focus on developing and psychometrically validating DCF-specific instruments while exploring whether existing measures, including the Zoom Exhaustion and Fatigue Scale (Fauville et al., 2021; Simbula et al., 2023) and the Digital Communication Empathy Scale (Collins et al., 2024), can be adapted to assess aspects of digitally mediated compassionate care. Cross-cultural validation and measurement invariance should also be established.

Methodologically, the current evidence remains dominated by cross-sectional and concept-development studies. Longitudinal, mixed-methods, intervention, and experience-sampling studies are needed to examine how Digital Compassion Fatigue and Digital Empathy develop over time and respond to organizational or educational interventions.

Operational priorities include prospective multicenter cohorts to test temporal ordering among digital workload, Digital Empathy, DCF-related symptoms, burnout, and turnover intention; psychometric studies using exploratory and confirmatory factor analysis, test-retest reliability, convergent and discriminant validity, and measurement invariance for a DCF instrument; cluster-randomized or stepped-wedge trials of workflow redesign, protected recovery time, digital-communication training, or AI-supported documentation; and mixed-methods or experience-sampling studies linking momentary digital demands with empathy, fatigue, therapeutic presence, and patient-reported relational quality. Priority outcomes should include clinician wellbeing, perceived empathy, therapeutic alliance, digital workload, usability, patient trust, safety, retention, and implementation outcomes (Abou Hashish and Alnajjar, 2025; Abou Hashish, 2025; Yildirim et al., 2026; Fauville et al., 2021; Simbula et al., 2023; Byrne, 2025; Wiljer et al., 2025; Collins et al., 2024).

Future research should also examine emerging technologies, including generative artificial intelligence, conversational agents, virtual reality, and other immersive digital health applications, with particular emphasis on human–AI collaboration and its effects on compassionate care. Educational research should evaluate curricula designed to strengthen Digital Empathy and digital communication competencies, whereas organizational research should investigate leadership strategies, implementation approaches, workflow redesign, and organizational interventions that promote compassionate digital practice.

Finally, greater disciplinary and geographical diversity is needed. Future studies should extend beyond nursing to include physicians, psychologists, social workers, allied health professionals, and other healthcare disciplines while increasing representation from underrepresented regions and healthcare systems (Table 7).

**Table 7:** Evidence gaps and future research priorities.

Domain	Evidence gap	Priority
Conceptual	Establish consensus definitions and clarify distinctions among Digital Compassion Fatigue, burnout, technostress, secondary traumatic stress, and traditional compassion fatigue.	High
Measurement	Develop and psychometrically validate Digital Compassion Fatigue-specific instruments; evaluate adaptation of existing digital empathy and fatigue measures.	High
Methodological	Conduct longitudinal, mixed-methods, intervention, and experience-sampling studies to investigate temporal relationships and intervention effectiveness.	High
Integration	Examine Digital Compassion Fatigue and Digital Empathy simultaneously within unified conceptual and analytical models.	High
Artificial intelligence	Investigate the effects of generative AI, conversational agents, and human–AI collaboration on compassionate care and clinician well-being.	Moderate
Leadership and organizations	Evaluate organizational interventions, leadership strategies, workflow redesign, and human-centered implementation approaches that support compassionate digital care.	Moderate
Education	Evaluate Digital Empathy curricula, simulation-based learning, and digital professionalism education across healthcare professions.	Moderate
Disciplinary diversity	Expand research beyond nursing to physicians, psychologists, social workers, allied health professionals, and interdisciplinary healthcare teams.	Moderate
Global representation	Increase evidence from low- and middle-income countries and underrepresented healthcare systems through multinational collaborative research.	Moderate

Priorities were determined through qualitative interpretation of the mapped evidence and are intended to guide future research rather than represent a validated ranking system.

### 4.11 Strengths and limitations

This review is, to our knowledge, the first to synthesize evidence on Digital Compassion Fatigue and Digital Empathy within a single conceptual framework across digitally mediated healthcare. By integrating concept analyses, empirical studies, evidence syntheses, theoretical papers, and implementation literature, the review provides a comprehensive overview of the current state of knowledge while proposing an evidence-informed Human-Centered Clinical Framework that links digital healthcare environments, adaptive resources, organizational conditions, and clinician outcomes.

Several limitations should be acknowledged. First, the available evidence is heterogeneous and is dominated by concept analyses, cross-sectional studies, and narrative or secondary syntheses, limiting causal inference. Second, DCF remains an emerging construct with no validated DCF-specific measurement instrument, restricting direct empirical comparison across studies. Third, the evidence is concentrated in nursing and in North American and European settings. This disciplinary and geographical imbalance may privilege digital infrastructures, staffing models, professional roles, communication norms, and resource conditions typical of higher-income health systems, limiting transferability to other professions and to low- and middle-income settings. Fourth, restricting inclusion to English-language publications may have underrepresented culturally specific understandings of empathy, compassion, digital work, and relational care and may partly explain the observed geographical concentration. Fifth, gray literature was not searched systematically. This may have increased publication bias by underrepresenting implementation reports, negative findings, local evaluations, and emerging evidence from settings where peer-reviewed publication is less accessible. Sixth, no formal critical appraisal was undertaken, consistent with the mapping purpose of the scoping review; consequently, the synthesis describes the range of available evidence without grading its certainty. These limitations mean that the proposed relationships and framework should not be interpreted as universally transferable or causally established.

The proposed Human-Centered Clinical Framework should therefore be regarded as an evidence-informed conceptual model that requires empirical testing across diverse healthcare settings, professions, and cultural contexts.

## 5 Conclusion

Digital Compassion Fatigue and Digital Empathy are best understood from the current evidence as related but distinct constructs situated within the same digitally transformed healthcare environment. Digital Empathy reflects the adaptation of empathic practice to technology-mediated encounters, whereas DCF remains a proposed technology-shaped form of compassion-related depletion that requires further empirical validation.

This review integrates the emerging literature on Digital Compassion Fatigue and the more established evidence on Digital Empathy into a unified conceptual perspective. Building on the synthesis of 41 evidence sources, it proposes an evidence-informed Human-Centered Clinical Framework that explains how digital healthcare environments, technology-related demands, individual adaptive resources, and organizational conditions interact to influence clinician wellbeing, patient relationships, and healthcare system outcomes.

The findings emphasize that sustainable digital transformation requires more than technological innovation. Equal attention should be given to compassionate care, clinician wellbeing, organizational support, and human-centered technology design. Future research should prioritize the development of validated Digital Compassion Fatigue measurement instruments, empirical evaluation of the proposed framework, longitudinal and intervention studies, and broader multidisciplinary research to advance compassionate, sustainable, and human-centered digital healthcare.

## Data Availability

The original contributions presented in the study are included in the article/Supplementary material, further inquiries can be directed to the corresponding author/s.
