# Supplementary material for: Digital compassion fatigue and digital empathy in healthcare: mapping the evidence and informing a human-centered clinical framework
Source: Front Psychol. 2026 Sep 14;17:1959352. doi: 10.3389/fpsyg.2026.1959352 (PMC13617115; doi:10.3389/fpsyg.2026.1959352)
Supplement: Supplementary file 1 [file Supplementary_file_1.docx]

**PRISMA-ScR Checklist**

*Supplementary File S1 — Companion to: Digital Compassion Fatigue and Digital Empathy in Healthcare: Mapping the Evidence and Informing a Human-Centered Clinical Framework*

*Completed in accordance with the PRISMA Extension for Scoping Reviews (PRISMA-ScR) checklist (Tricco et al., 2018, Annals of Internal Medicine).*

| **Section/Topic** | **Item #** | **Checklist Item** | **Reported in Section** |
| --- | --- | --- | --- |
| **TITLE** |  |  |  |
| Title | 1 | Identify the report as a scoping review. | Title page |
| **ABSTRACT** |  |  |  |
| Structured summary | 2 | Provide a structured summary that includes (as applicable): background, objectives, eligibility criteria, sources of evidence, charting methods, results, and conclusions. | Abstract |
| **INTRODUCTION** |  |  |  |
| Rationale | 3 | Describe the rationale for the review in the context of what is already known. | Section 1, Introduction |
| Objectives | 4 | Provide an explicit statement of the questions and objectives being addressed, with reference to key elements (e.g., population/participants, concepts, and context). | Section 1.1, Review objectives; Main review question |
| **METHODS** |  |  |  |
| Protocol and registration | 5 | Indicate whether a review protocol exists, state if and where it can be accessed, and if available, provide registration information. | Section 2.2, Protocol and registration |
| Eligibility criteria | 6 | Specify characteristics of the sources of evidence used as eligibility criteria (e.g., years considered, language, publication status), and provide a rationale. | Section 2.5, Eligibility criteria (PCC); Table 1 |
| Information sources | 7 | Describe all information sources in the search (e.g., databases with dates of coverage and contact with authors to identify additional sources), as well as the date the most recent search was executed. | Section 2.3, Information sources; Supplementary Material S1–S1a |
| Search | 8 | Present the full electronic search strategy for at least one database, including any limits used, such that it could be repeated. | Section 2.4, Search strategy; Supplementary Material S2 |
| Selection of sources of evidence | 9 | State the process for selecting sources of evidence (i.e., screening and eligibility) included in the scoping review. | Section 2.7, Selection process |
| Data charting process | 10 | Describe the methods of charting data from the included sources of evidence (e.g., calibrated forms or forms that have been tested by the team before their use, and whether data charting was done independently or in duplicate) and any processes for obtaining and confirming data from investigators. | Section 2.8, Data extraction; Supplementary Material S7 |
| Data items | 11 | List and define all variables for which data were sought and any assumptions and simplifications made. | Section 2.8, Data extraction; Supplementary Material S7, Table S7.1 |
| Critical appraisal of individual sources of evidence | 12 | If done, provide a rationale for conducting a critical appraisal of included sources of evidence; describe the methods used and how this information was used in any data synthesis. | Section 2.10, Risk of bias (not undertaken; rationale provided) |
| Synthesis of results | 13 | Describe the methods of handling and summarizing the data that were charted. | Section 2.9, Data synthesis |
| **RESULTS** |  |  |  |
| Selection of sources of evidence | 14 | Give numbers of sources of evidence screened, assessed for eligibility, and included, with reasons for exclusion at each stage, ideally with a flow diagram. | Section 3.1, Search results; Figure 1 |
| Characteristics of sources of evidence | 15 | For each source of evidence, present characteristics for which data were charted and provide citations. | Section 3.2–3.8; Table 2 |
| Critical appraisal within sources of evidence | 16 | If done, present data on critical appraisal of included sources of evidence. | Not applicable — critical appraisal not undertaken (Section 2.10) |
| Results of individual sources of evidence | 17 | For each included source of evidence, present the relevant data that were charted that relate to the review questions and objectives. | Table 2; Sections 3.2–3.8 |
| Synthesis of results | 18 | Summarize and/or present the charting results as they relate to the review questions and objectives. | Sections 3.2–3.8; Tables 2–7; Discussion |
| **DISCUSSION** |  |  |  |
| Summary of evidence | 19 | Summarize the main results (including an overview of concepts, types of evidence, and populations) and link to the review questions and objectives. | Section 4, Discussion |
| Limitations | 20 | Discuss the limitations of the scoping review process. | Section 4.10, Strengths and limitations |
| Conclusions | 21 | Provide a general interpretation of the results with respect to the review questions and objectives, as well as potential implications and/or next steps. | Section 5, Conclusion |
| **FUNDING** |  |  |  |
| Funding | 22 | Describe sources of funding for the included sources of evidence, as well as sources of funding for the scoping review. Describe the role of the funders of the scoping review. | Funding statement |

*Note. Item 12 and the corresponding results item (16) are reported as “not applicable” because, consistent with JBI guidance for scoping reviews, critical appraisal of individual sources of evidence was not undertaken (Section 2.10); this is a standard and accepted approach for scoping reviews, whose objective is to map the breadth and characteristics of available evidence rather than evaluate intervention effectiveness.*
